# Proteome-wide Mendelian randomization identifies natriuretic peptide-B and novel proteins as potential regulators of pulse pressure in humans

**DOI:** 10.1161/JAHA.124.037596

**Published:** 2025-08-12

**Authors:** Marie-Joe Dib, Devendra Meena, James Yarmolinsky, Joe David Azzo, Oday Salman, Hamed Tavolinejad, Sushrima Gan, Cameron Beeche, Bianca Pourmussa, Dipender Gill, Stephen Burgess, Julio A. Chirinos

**Affiliations:** 1 Division of Cardiovascular Medicine, Hospital of the University of Pennsylvania, Philadelphia PA; 2 University of Pennsylvania Perelman School of Medicine, Philadelphia, PA; 3 Department of Epidemiology and Biostatistics, School of Public Health, Imperial College London, UK; 4 Department of Pediatrics (Cardiology) and Cardiovascular Institute, Stanford University; 5 MRC Integrative Epidemiology Unit, University of Bristol, Bristol, UK; 6 Department of Public Health and Primary Care, University of Cambridge, Cambridge, UK

**Keywords:** Pulse pressure, arterial stiffness, proteomics, Mendelian randomization

## Abstract

**Introduction:**

Large artery stiffness (LAS) significantly contributes to cardiovascular morbidity and mortality and is characterized by increased pulse pressure (PP). The biology underlying LAS in humans remains incompletely understood.

**Methods:**

We investigated associations between PP and circulating levels of 2,941 proteins among 53,016 UK Biobank participants. Analyses were adjusted for age, sex, mean arterial pressure (MAP), body mass index (BMI) and stroke volume (SV). Interaction analyses assessed the effect modification by sex on these relationships. We evaluated causal associations between plasma protein levels and PP, using inverse-variance weighted Mendelian randomization (MR) as the main analysis, and Bayesian colocalization as a sensitivity analysis. A 5% false discovery rate (FDR) threshold was used to account for multiple comparisons.

**Results:**

Measured levels of 871 proteins were significantly associated with PP when adjusting for age, sex, MAP and BMI, and 61 remained significantly associated after further adjusting for SV. Top associations included natriuretic peptide B (NPPB), thrombospondin-2, paraoxonase-2, and sclerostin. Genetic analyses indicated that genetically predicted levels for 16 proteins were significantly associated with PP after FDR correction, including fibroblast growth factor 5 (FGF5; β_IVW_ per SD change in protein levels=0.47, 95% CI=0.34,0.61), NPPB (β_IVW_=-1.40; 95% CI=-1.85, -0.95), insulin like growth factor binding 3 (IGFBP3; β_IVW_=-1.143; 95% CI=-1.57, -0.71), and furin (β_IVW_=1.31; 95% CI=0.88,1.73).

**Conclusions:**

Using complementary epidemiological approaches to triangulate findings, our study identifies novel proteins with a putative causal effect on PP. Notably, our findings identify NPPB with high statistical confidence. This may have potentially impactful implications given the current availability of FDA-approved medications to boost NPPB effects.

## Non-standard Abbreviations and Acronyms

ABO: Alpha 1-3-N-Acetylgalactosaminyltransferase and alpha 1-3-galactosyltransferase
ADM: adrenomedullin
ADAMTS8: A disintegrin and metalloproteinase with thrombospondin motifs 8
ANGPT2: angiopoietin-2
BCAN: brevican
BMI: body mass index
COL4A1: collagen type IV alpha 1 chain
COMP: cartilage oligomeric matrix protein
CXCL10: C-X-C motif chemokine ligand 10
F13B: coagulation factor XIII B chain
FDR: false discovery rate
FGF5: fibroblast growth factor 5
GIP: glucose-dependent insulinotropic polypeptide
IGFP3: insulin like growth factor binding 3
IL1B: interleukin-1 beta
ITGAL: integrin alpha-L
IVW: inverse-variance weighted
LAS: large artery stiffness
LTBP2: latent transforming growth factor beta binding protein 2
MAP: mean arterial pressure
MOG: myelin oligodendrocyte glycoprotein
OMG: oligodendrocyte myelin glycoprotein
NPPB: natriuretic peptide B
PP: pulse pressure
PWV: pulse wave velocity
MR: Mendelian randomization
PDGF: platelet-derived growth factor
PLA2G7: phospholipase A2 group VII
PLAT: plasminogen activator, tissue type
SNP: single nucleotide polymorphism
SOST: sclerostin
SV: stroke volume
SD: standard deviation
REN: renin
SPOCK1: SPARC/Osteonectin, Cwcv And Kazal Like Domains Proteoglycan 1
THBS2: thrombospondin-2
TNFSF10: TNF superfamily member 10
TNFSF12: tumor necrosis factor ligand superfamily member 12
UBE2L6: ubiquitin/ISG15-conjugating enzyme E2 L6

## Introduction

The increasing prevalence of the aging population worldwide is an unprecedented demographic phenomenon expected to drive a growing wave of age-related healthcare and economic burden globally^1–3^. Aging and multiple pathological conditions that are acquired over the lifetime lead to increased risk of large artery stiffness (LAS)^4^. A clinical consequence of LAS is an increased systolic blood pressure during left ventricular ejection and a decreased diastolic pressure during diastolic runoff, resulting in a higher pulse pressure (PP)^5^. Increased LAS and PP lead to excess pulsatility in the microvasculature, particularly in low-resistance target organs such as the kidney and the brain^6^, and impacts left ventricular afterload and coronary perfusion pressure via its effects on pulsatile load.^5^ As such, a higher PP, which is a direct consequence of increased LAS, has been shown to be associated with target organ damage (including worsening of kidney function,^7–10^ cognitive decline^6,9^ and new-onset diabetes ^11–13^), and is established as an independent risk factor for cardiovascular disease and all-cause mortality^14^. Understanding the molecular basis of PP elevation may provide novel insights underlying LAS in humans, thereby leading to the improved diagnosis and treatment of LAS and its downstream clinical manifestations.

Genome-wide association of plasma proteomics, applied among large population cohorts, ^15–18^ has paved the way for enhanced insights into the genetic regulation of the plasma proteome, providing a unique opportunity to evaluate the causal effects of individual proteins on the development and progression of various conditions. Mendelian randomization (MR) is a powerful method that enables the identification of causal effects in humans, overcoming some limitations of traditional observational studies such as residual confounding and reverse causality. MR leverages the naturally randomized allocation of genetic variants among the population as instrumental variables, analogous to treatment allocation in a randomized controlled trial. Under certain assumptions, this approach estimates the causal effects of exposures on outcomes.^19^ Pairing plasma proteomics with human genetics for causal inferencing can provide important insights on the putative causal relationship between the human proteome and various traits and conditions. However, studies investigating the proteome in relation to the manifestation of LAS, including PP, have not been conducted.

In this study, we aimed to (1) evaluate the relationship between plasma proteins and PP in middle-aged adults from the UK Biobank, (2) assess the putative causal associations of specific plasma proteins on PP using MR and Bayesian colocalization analysis.

## Methods

**Figure 1:**
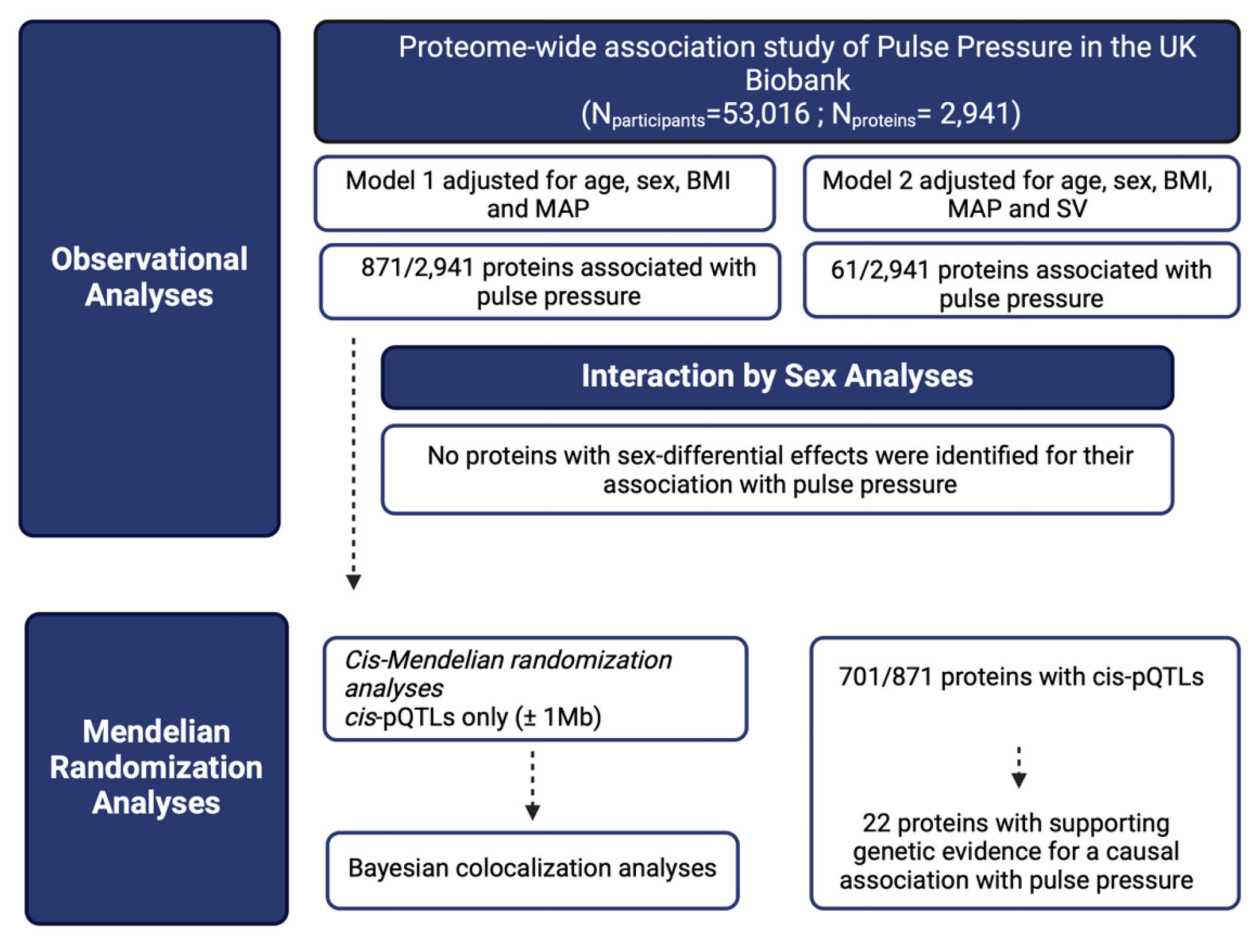
Proteome-wide association study of 2,941 proteins for pulse pressure in the UK Biobank. **Abbreviations:** BMI, body mass index; MAP, mean arterial pressure; pQTLs, protein quantitative trait loci; SV, stroke volume.

The study design is illustrated in Figure 1. This study is reported using the Strengthening the Reporting of Observational Studies in Epidemiology-Mendelian randomization (STROBE-MR) guidelines ^20,21^ (https://www.strobe-mr.org) (Supplemental Data). The UK Biobank study was approved by the UK’s North West Multi‐Centre Research Ethics Committee. All participants provided written informed consent.

### Study population

The UK Biobank is a large population-based prospective study, established to allow investigation of the genetic and non-genetic determinants of the diseases of middle and old age. Participants aged 40-69 years at the time of study enrolment and were recruited from 22 assessment centers across the United Kingdom from 2006 to 2010. Participants provided informed consent at recruitment. Details of the cohort have been previously described. ^22^ Data accession was facilitated under UK Biobank application number 81032.

### Proteomics measurements

Proteomic profiling was performed on blood plasma samples collected from 53,016 UK Biobank participants using the antibody-based Olink Explore 3072 PEA. This platform measures 2,923 unique proteins across the Cardiometabolic, Inflammation, Neurology and Oncology panels. Details on NPX processing and quality control procedures have been previously described.^23^

### Proteomics Association Analyses

In proteome-wide analyses, we assessed the relationship between individual plasma protein biomarkers and PP using linear regression models. Considering that mean arterial pressure (MAP) and body mass index (BMI) are both associated with PP, ^24,25^ we conducted regression analyses in Model 1 including age, sex, MAP and BMI as covariates. Given that stroke volume (SV) can impact PP independently of LAS, we also built models (Model 2) that further adjusted for SV measured via cardiac magnetic resonance imaging. Estimates for all proteins are standardized (expressed per SD increase, or 1-point increase in the *z*-score). We performed linear regression interaction analyses adjusted for age, MAP, and BMI to assess the effect modification by sex on the relationship between circulating protein levels and PP. Correction for multiple testing was performed using the false discovery rate (FDR) Benjamini-Hochberg method.^26^ Pathway enrichment analyses are described in detail in Supplemental Methods.

### Genetic Analyses

#### Selection of genetic instruments

Using proteomics data from the UK Biobank (N= 53,016), we first identified *cis* (within ± 1Mb of the protein-encoding region)- acting protein quantitative trait loci (pQTLs) for circulating protein levels of each of the identified proteins in the previous analyses. Proteins were analyzed if pQTLs with association *p*<5x10^-8^ were available. We restricted our analyses to *cis*-pQTLs as they are more likely to have protein-specific effects than *trans*-pQTLs.^27^ The use of *cis*-pQTLs also ensures that the MR assumption of ‘no horizontal pleiotropy’ (*i.e*. that the instrument is not related to the outcome other than via the exposure of interest) is not violated. Independent genetic instruments were selected using a clumping threshold r^2^ < 0.001 using a random sample of 10,000 individuals of White British ancestry in the UK Biobank as reference panel. We estimated the strength of each instrument by calculating the F statistic. A F statistic > 10 was indicative of adequate instrument strength.^28^

### Two-sample Mendelian randomization

We conducted MR analyses to estimate the effect of genetically predicted plasma protein levels on PP. MR is a statistical approach that uses genetic variants as instrumental variables to proxy the causal effects of an exposure on an outcome of interest. Three fundamental assumptions underpin the effective utilization of genetic instruments to facilitate robust MR experiments. Firstly, the genetic instrument(s) must be associated with the exposure of interest (*i.e*., the relevance assumption). Secondly, no confounding factors influence the association between the genetic instrument(s) and outcomes of interest (*i.e*., the independence assumption). Thirdly, the genetic instrument is only related to the outcome via the exposure, ensuring the absence of pleiotropic effects that may bias MR estimates (*i.e*., the exclusion restriction assumption).^19^ For our outcome dataset, we defined and calculated PP as the difference between systolic and diastolic blood pressure for 230,422 UK Biobank participants and conducted a genome-wide association study of PP, excluding participants with proteomics data to avoid sample overlap in MR analyses, as described in detail in Supplemental Methods. As a sensitivity analysis, we then conducted the same analyses while accounting for antihypertensive medication use. Specifically, we adjusted systolic blood pressure by adding 10 mmHg and diastolic blood pressure by adding 5 mmHg when deriving PP in participants taking antihypertensive medication. We used a Wald ratio when one single nucleotide polymorphism (SNP) was available for MR,^29^ and inverse-variance weighted (IVW) MR when more than 1 SNP was available.^30^ We estimated the I^2^ statistic to detect heterogeneity among the MR estimates obtained from multiple genetic variants. We applied adjustments for multiple comparisons using the FDR method.^26^ We report estimated β and 95% confidence intervals (CI) for the putative effects of these proteins on PP. β values represent the changes in PP in mmHg for every standard deviation (SD) change in genetically predicted plasma protein levels.

We conducted Bayesian colocalization as a sensitivity analysis to confirm that each protein and PP shared the same causal variant. This is described in further detail in Supplemental Methods.

## Results

General characteristics of study participants that had proteomics data in the UK Biobank cohort (N=53,016) are shown in Table 1.

### Associations between measured protein levels and PP

**Figure 2:**
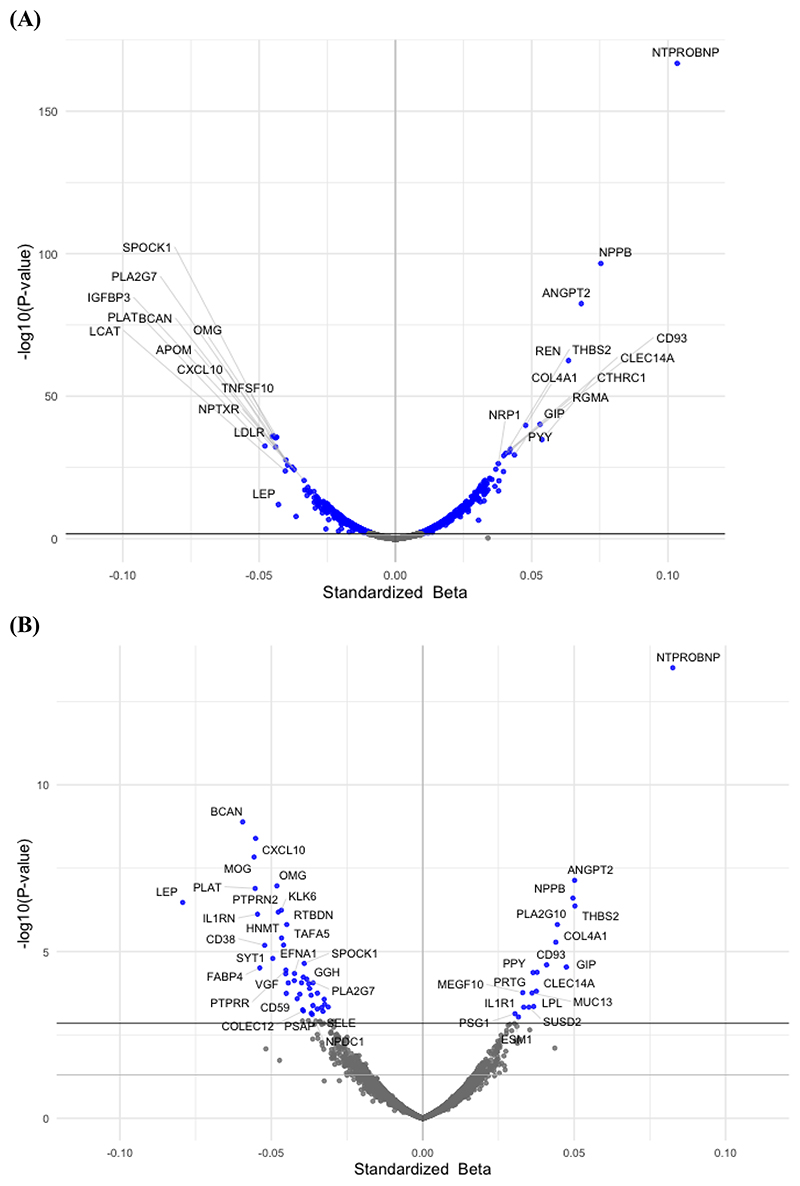
Volcano plot representing proteins significantly associated with pulse pressure in observational analyses for (A) the model adjusted for age, sex, body mass index (BMI), and mean arterial pressure (MAP); and (B) the model adjusted for age, sex, body mass index (BMI), mean arterial pressure (MAP), and stroke volume (SV). The plot shows β estimates against the false discovery rate (FDR) corrected log-10 *p* value, to better visualize the importance of each biomarker in order of significance. The dashed line represents the 5% FDR alpha threshold. **Abbreviations:** ANGPT2, Angiopoietin-2; APOM, Apolipoprotein M; BCAN, Brevican; CD38, Cluster of Differentiation 38; CD46, Cluster of Differentiation 46; CD59, Cluster of Differentiation 59; CD93, Cluster of Differentiation 93; CLEC14A, C-Type Lectin Domain Family 14 Member A; COL4A1, Collagen Type IV Alpha 1 Chain; CTHRC1, Collagen Triple Helix Repeat Containing 1; CXCL10, C-X-C Motif Chemokine Ligand 10; EFNA1, Ephrin-A1; FABP4, Fatty Acid Binding Protein 4; GGH, Gamma-Glutamyl Hydrolase; GIP, Gastric Inhibitory Polypeptide; HNMT, Histamine N-Methyltransferase; IGFBP3, Insulin-Like Growth Factor Binding Protein 3; IL1B, Interleukin 1 Beta; IL1R1, Interleukin 1 Receptor Type 1; IL1RN, Interleukin 1 Receptor Antagonist; KLK6, Kallikrein-Related Peptidase 6; LCAT, Lecithin-Cholesterol Acyltransferase; LEP, Leptin; LDLR, Low-Density Lipoprotein Receptor; LPL, Lipoprotein Lipase; MEGF10, Multiple EGF-Like Domains 10; MOG, Myelin Oligodendrocyte Glycoprotein; MUC13, Mucin 13; NPDC1, Neural Proliferation Differentiation and Control 1; NPPB, Natriuretic Peptide B; NPROBNP, N-Terminal Prohormone of Brain Natriuretic Peptide; NPTXR, Neuronal Pentraxin Receptor; NRP1, Neuropilin 1; OMG, Oligodendrocyte Myelin Glycoprotein; PECAM1, Platelet and Endothelial Cell Adhesion Molecule 1; PLA2G7, Phospholipase A2 Group VII; PLA2G10, Phospholipase A2 Group X; PLAT, Plasminogen Activator Tissue Type; PLATBCAN, Plasminogen Activator Tissue Type and Brevican; PRG, Proteoglycan; PRKAB1, Protein Kinase AMP-Activated Non-Catalytic Subunit Beta 1; PRTG, Protogenin; PSG1, Pregnancy-Specific Beta-1-Glycoprotein 1; PSAP, Prosaposin; PTPRN2, Protein Tyrosine Phosphatase Receptor Type N2; PTPRR, Protein Tyrosine Phosphatase Receptor Type R; PYY, Peptide YY; REN, Renin; RGMA, Repulsive Guidance Molecule A; RTBDN, Retina And Optic Nerve Development Protein; SELE, Selectin E; SLC9A3R2, Solute Carrier Family 9 Member 3 Regulator 2; SMOC2, SPARC Related Modular Calcium Binding 2; SPARC, Secreted Protein Acidic and Rich in Cysteine; SPOCK1, SPARC/Osteonectin, Cwcv and Kazal Like Domains Proteoglycan 1; SUSSD2, Sushi Domain Containing 2; SYT1, Synaptotagmin 1; TAFA5, TAFA Chemokine Like Family Member 5; THBS2, Thrombospondin 2; TNFSF10, Tumor Necrosis Factor Superfamily Member 10; VGF, VGF Nerve Growth Factor Inducible.

In regression models adjusted for age, sex, MAP and BMI (model 1), we found 871 out of 2,923 proteins to be significantly associated with PP. The β estimates and *P*-values are included in Table S1. A volcano plot showing the relationship between baseline plasma protein levels and PP is shown in Figure 2A.

The top proteins positively associated with PP included NT-proBNP, natriuretic peptide B (NPPB), angiopoietin-2 (ANGPT2), renin (REN), glucose-dependent insulinotropic polypeptide (GIP) and collagen type IV alpha 1 chain (COL4A1). The top proteins negatively associated with PP included SPARC/Osteonectin, Cwcv And Kazal Like Domains Proteoglycan 1 (SPOCK1), phospholipase A2 group VII (PLA2G7), TNF superfamily member 10 (TNFSF10), oligodendrocyte myelin glycoprotein (OMG), and plasminogen activator, tissue type (PLAT). A summary of top associations is shown in Table 2.

The top canonical pathways associated with PP included metabolic, inflammatory and tissue remodelling pathways, including the LXR/RXR activation pathway, the retinoid metabolism and transport pathway, granulocyte adhesion and diapedesis, inhibition of matrix metalloproteases, and glycosaminoglycan metabolism (Figure S1A).

In regression models further adjusted for SV (model 2), we found 61 out of 2,923 proteins to be significantly associated with PP. The β estimates and *P*-values are shown in Table S2. A volcano plot showing the relationship between plasma protein levels and PP is shown in Figure 2B. The top proteins positively associated with PP included NT-proBNP, ANGPT2, NPPB, thrombospondin-2 (THBS2), PLA2G10, and COL4A1. The top proteins negatively associated with PP included brevican (BCAN), C-X-C motif chemokine ligand 10 (CXCL10), myelin oligodendrocyte glycoprotein (MOG), OMG and PLAT (Table 2).

The top canonical pathways associated with PP included signalling by platelet-derived growth factor (PDGF), phospholipases, transcriptional regulation of white adipocyte differentiation, synthesis, secretion and diacylation of ghrelin, and collagen chain trimerization (Figure S1B).

### Sex-specific protein associations with PP

We formally tested for effect modification by sex across all protein associations with PP in 2 regression models using protein-sex interaction terms in regression models. We also conducted sex-stratified analyses to obtain sex-specific effect estimates for differentially associated proteins. After correction for multiple testing, 184 proteins exhibited sex-differential associations with PP in Model 1 (Table S3). Sex-stratified results for proteins are presented in Tables S4-S5. Volcano plots for sex-stratified analyses for multivariable models adjusting for age, BMI and MAP are depicted in Figure S2. However, when SV was added as a covariate to the regression models (Model 2), none of the proteins exhibited significant sex-differential associations with PP (Table S6). Sex-stratified results for proteins in Model 2 are shown in Tables S7-S8. Canonical pathway results are shown in Figure S3.

### Two-sample Mendelian randomization analyses

**Figure 3:**
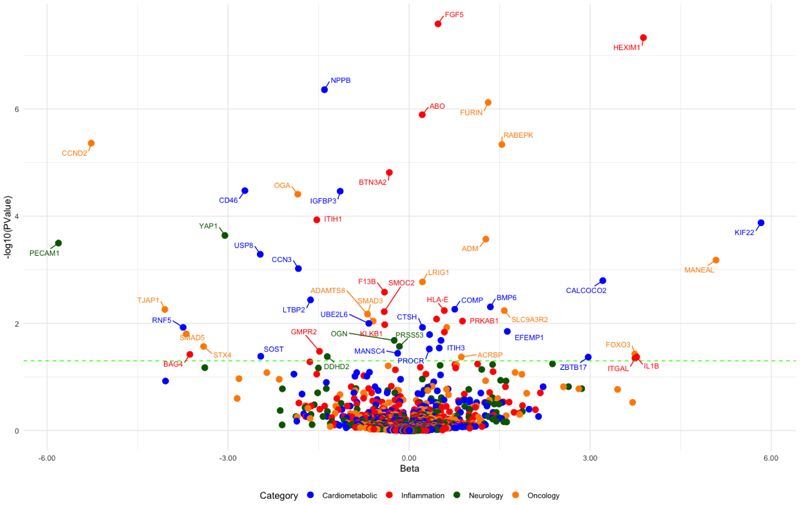
Volcano plot representing proteins significantly associated with pulse pressure in Mendelian randomization analyses. The plot shows β estimates against the false discovery rate (FDR) corrected log-10 *p* value, to better visualize the importance of each biomarker in order of significance. The dashed line represents the 5% FDR alpha threshold. **Abbreviations:** ABO, Alpha 1-3-N-Acetylgalactosaminyltransferase and alpha 1-3-galactosyltransferase; ADM, adrenomedullin; CD46, cluster of differentiation 46; EFEMP1, EGF-containing fibulin-like extracellular matrix protein 1; F13B, coagulation factor XIII B subunit; FGF5, fibroblast growth factor 5; FOXO3, Forkhead box O3; HLA-E, major histocompatibility complex, class I, E; IGFBP3, Insulin-like growth factor-binding protein 3; IL1B, Interleukin 1 beta; ITGAL, Integrin subunit alpha L; KIF22, kinesin family member 22; KLKB1, kallikrein B1; LTBP2, Latent Transforming Growth Factor Beta Binding Protein 2; MANEAL, Mannose Endo-alpha-1,2-Mannosidase Like; NPPB, natriuretic peptide B; OGN, Osteoglycin; PECAM1, Platelet and endothelial cell adhesion molecule 1; PRKAB1, Protein Kinase AMP-Activated Non-Catalytic Subunit Beta 1; PRSS53, serine protease 53; RABEPK, Rab9 Effector Protein with Kelch Motif; SLC9A3R2, Solute Carrier Family 9 Member 3 Regulator 2; SMAD3, SMAD Family Member 3; SMOC2, SPARC Related Modular Calcium Binding 2; SOST, sclerostin; SPARC, Secreted Protein Acidic and Rich in Cysteine; TJAP1, Tight Junction Associated Protein 1; UBE2L6, Ubiquitin Conjugating Enzyme E2 L6; USP8, Ubiquitin Specific Peptidase 8; YAP1, Yes Associated Protein 1; ZBTB17, Zinc Finger And BTB Domain Containing 17.

We conducted two-sample MR to investigate associations for all genetically predicted plasma proteins available in the Olink panel. Results are shown in Figure 3. Considering proteins that were significantly associated with PP from our previous observational analyses, 701 out of 871 had available *cis*-pQTLs for MR analyses. Genetically predicted levels for 22 proteins were significantly associated with PP after FDR correction (Table 3). Thirteen out of 22 had concordant directions of effect with estimates from measured levels. The top five proteins positively associated with PP included fibroblast growth factor 5 (FGF5), furin, Alpha 1-3-N-Acetylgalactosaminyltransferase and alpha 1-3-galactosyltransferase (ABO), adrenomedullin (ADM) and cartilage oligomeric matrix protein (COMP). The top five proteins negatively associated with PP included natriuretic peptide B (NPPB), CD46, insulin like growth factor binding 3 (IGFBP3), coagulation factor XIII B chain (F13B), and latent transforming growth factor beta binding protein 2 (LTBP2). MR results investigating the full panel of proteins and PP are presented in Table S9. Results were consistent in sensitivity analyses considering PP adjusted for antihypertensive medication use (Figure S4, Table S10).

**Figure 4:**
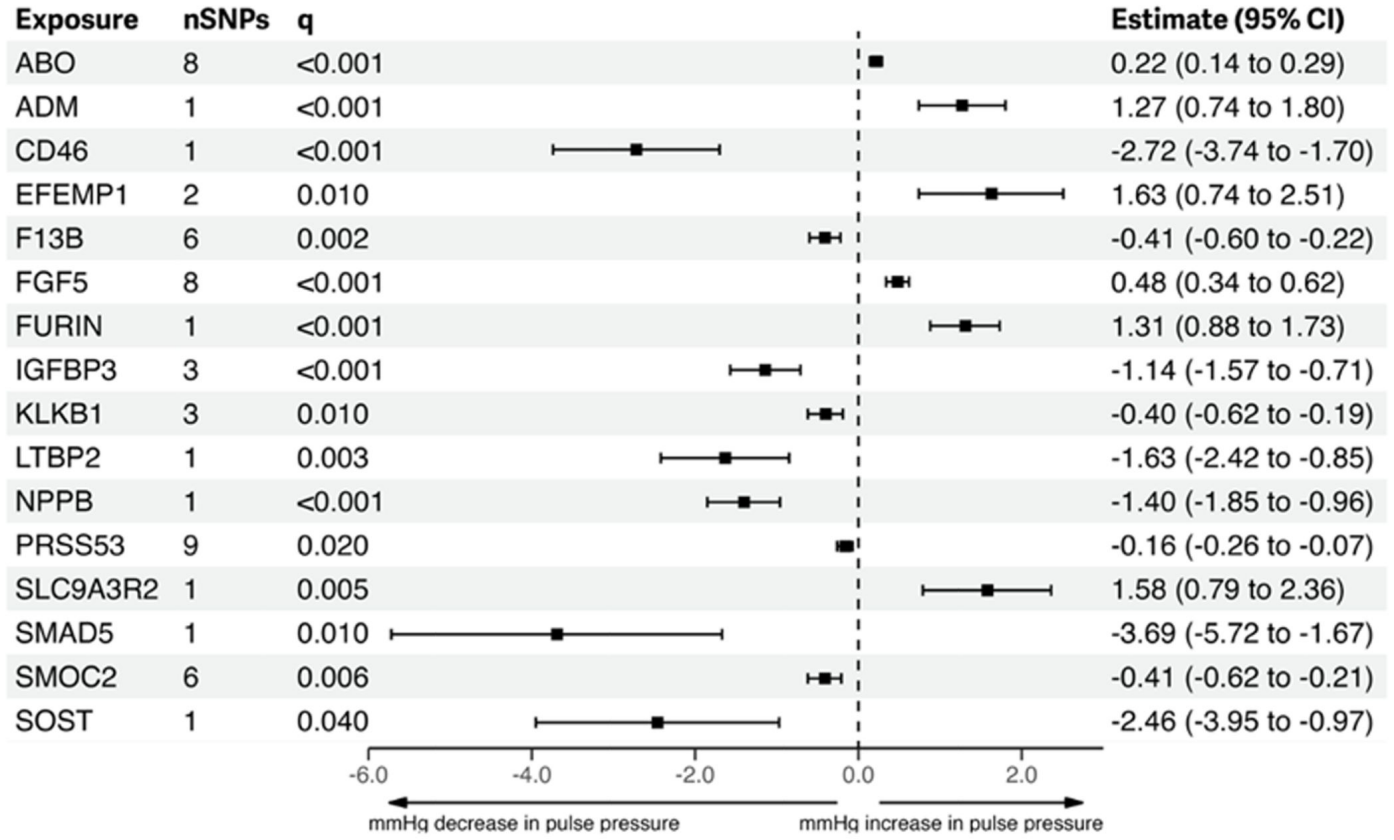
Two-sample MR effect estimates for association between 16 genetically instrumented circulating plasma protein levels and pulse pressure. Only associations between genetically predicted proteins and pulse pressure with statistically significant estimates (q<0.05) and with evidence of colocalization (posterior probability H_4_>0.50) are reported. Estimates and 95% confidence intervals are reported in mmHg per 1 SD increase in genetically predicted plasma protein levels. **Abbreviations:**ABO, Alpha 1-3-N-Acetylgalactosaminyltransferase and alpha 1-3-galactosyltransferase**;**ADM, adrenomedullin**;** CD46, cluster of differentiation 46; EFEMP1, EGF-containing fibulin-like extracellular matrix protein 1; F13B, coagulation factor XIII B subunit; FGF5, fibroblast growth factor 5; IGFBP3, Insulin-like growth factor-binding protein 3; KLKB1, kallikrein B1; LTBP2, Latent Transforming Growth Factor Beta Binding Protein 2; NPPB, natriuretic peptide B; PRSS53, serine protease 53; SLC9A3R2, ; SMAD5, SMAD Family Member 5; SMOC2, SPARC Related Modular Calcium Binding 2; SOST: sclerostin.

Colocalization analyses suggested shared causal genetic variants between each of the proteins and PP (posterior probability for shared causal variants H_4_>0.50), except for A disintegrin and metalloproteinase with thrombospondin motifs 8 (ADAMTS8), cartilage oligometric matrix protein (COMP), interleukin-1 beta (IL1B), integrin alpha-L (ITGAL), tumor necrosis factor ligand superfamily member 12 (TNFSF12) and ubiquitin/ISG15-conjugating enzyme E2 L6 (UBE2L6) (Table S11). Sensitivity analyses were indicative of support for the shared causal variant hypothesis at varying values of *p_12_* (Figure S5). Estimates for 16 proteins with evidence from MR and colocalization analyses supporting potentially causal associations with PP are shown in Figure 4. For all these proteins, sensitivity analyses were in support of the shared causal variant hypothesis at any value of the *p_12_* prior (Figure S5).

## Discussion

This is the first study to broadly assess proteomic associations of PP in an older community-based population sample. Among 53,016 UK Biobank participants, we identified 871 proteins significantly associated with PP, including NT-proBNP, NPPB, ANGPT2, REN, GIP, PLA2G7, SPOCK1, TNFSF10, OMG, PLAT and COL4A1. Many of these proteins exhibit high biologic plausibility based on their known functions. Furthermore, we performed genetic analyses, providing supporting evidence of a causal effect of 16 proteins on PP, including the peptide NPPB, a well know hemodynamic regulator which can be enhanced with currently available therapeutic agents. Adjusted analyses did not identify sex-specific effects, suggesting that these associations are present in both males and females. Our findings provide novel insights into the mechanisms of increased PP and LAS and prioritize candidate therapeutic targets for intervention.

### Associations between NPPB, NT-proBNP and pulse pressure

Our findings identified NPPB as the most significant protein to be associated with PP in both observational and MR analyses. We note that the association between genetically predicted NPPB and PP was inverse, whereas the association between NT-proBNP and PP was direct. This is readily explained by the generally favorable biologic actions of NPPB, which contrast with the adverse epidemiologic and clinical significance of elevated NT-proBNP levels (which usually coincide with conditions that increase the stress on cardiomyocytes). NT-proBNP is a fragment of proBNP, the precursor molecule of BNP, and has been previously associated with adverse cardiovascular outcomes. A previous proteome-wide association study by Azzo *et al* showed that increased NT-proBNP levels in heart failure with preserved ejection fraction are associated with pathways related to fibrosis and matrix metalloproteinases, which are known regulators of arterial stiffening.^31,32^ Several studies previously investigated the role of BNP and NT-proBNP in arterial stiffness in smaller studies.^33–39^ Minoru *et al* showed that PP and brachial-ankle pulse wave velocity (PWV) were significantly positively associated with plasma BNP levels independent age in a healthy Japanese population.^33^ In the Rotterdam study population, Joost *et al* demonstrated that plasma NT-proBNP levels were significantly positively correlated with carotid-femoral PWV.^39^ Moreover, in a renal transplant population, plasma NT-proBNP level was a significant positive predictor of cardio-ankle vascular index independent of conventional cardiovascular risk factors.^2^ Similarly, Qing *et al* also found NT-proBNP to be significantly associated with brachial-ankle PWV in a Takayasu arteritis patient population.^36^ In the Framingham Heart study population, another multivariable regression done by Daniel *et al*, exhibited a significant correlation of BNP with carotid-femoral and carotid-radial PWV as well as carotid PP across both men and women independent of conventional cardiovascular risk factors including prior CVD.^35^ BNP was negatively associated with carotid-femoral PWV in women, but positively associated with it in men, indicating a differential sex-specific association, in accordance with our observational findings which revealed a sex-differential association of NPPB and NT-proBNP with PP, with a positive relationship observed for NT-proBNP in males. However, it is important to note that: (1) PP is highly dependent on stroke volume, which is known to differ between men and women; (2) BNP is a well-known regulator of intravascular volume, which can impact stroke volume, and (3) Observational associations cannot be taken as surrogates of causal effects. We performed adjustment for SV measured via cardiac magnetic resonance imaging, which represents the gold standard method for stroke volume measurements. Importantly, although the association between NT-proBNP and NPPB and PP persisted in these analyses, sex differences in these associations were no longer present when stroke volume was considered, indicating that these depend on sex differences in stroke volume. We also performed MR and genetic colocalization analyses to assess the potential causal effect of NPPB on PP. In agreement with the known beneficial effects of natriuretic peptide B on the vasculature, we observed a negative association between genetically predicted NPPB and PP. This suggests that the positive observational association represents a counter-regulatory association which may be triggered by the effects of pulse pressure and pulsatile afterload on the left ventricle, leading to an increased release of NPPB. These findings illustrate the utility of MR and genetic colocalization analyses to reduce or eliminate confounding, allowing for a better assessment of potentially causal associations.

Our identification of the association of genetically predicted NPPB and PP, suggesting a potentially causal effect, has important implications, given the current availability of FDA-approved medications to boost NPPB effects in humans, based on neprilysin inhibition. Interestingly, Mitchell *et al*. demonstrated that treatment with sacubitril-valsartan, compared to enalapril, resulted in a significant reduction of aortic characteristic impedance (a key hemodynamic determinant of PP influenced by aortic root stiffness and size) as well as a reduction in brachial PP among participants with heart failure with reduced ejection fraction.^37^ Whereas Peder *et al* showed that sacubitiril-valsartan did not lead to significant changes in plasma concentrations of BNP and NT-proBNP in participants with heart failure with reduced ejection fraction, it did show a marked increase in urinary concentration of cyclic-guanosine monophosphate, which may be indicative of increased natriuretic peptide signaling.^38^ Hence, the difference in effect of Sacubitril-Valsartan as compared to enalapril on aortic root characteristic impedance and PP shown by Mitchell *et al* may be mediated by enhanced natriuretic peptide signalling. Further studies should be performed regarding the role of NPPB as a regulator of PP, and NPPB as candidate therapeutic target to ameliorate LAS and reduce PP.

### Other proteomic associations with PP

We also found associations between genetically predicted levels of multiple proteins and PP, supporting putative causal associations. We identified several novel proteins associated with PP in humans, including ABO, NPPB, IGFBP3, CD46, EFEMP1, F13B, FGF5, Furin, LTBP2, SOST, PRSS53, SMOC2 and SMAD5.

IGFBP3 was negatively associated with brachial PP in our analyses. IGFBP-3 is a key protein in the IGF pathway and plays a role in regulating various biological actions of the IGFs, including cellular proliferation, differentiation, and increased metabolic activity.^40^ There is conflicting literature on the role of IGFBP3 in the progression of arterial stiffness and disease. A genome-wide study of PP nested in the Framingham Heart Study (N=8,478) revealed a connection between PP and genetic loci associated with IGFBP3, suggesting a potential role for IGFBP3 in vascular stiffness.^41^ Moreover, IGFBP-3 was found to act as a vascular protective protein following vascular injury, promoting revascularization by increasing eNOS expression in human endothelial progenitor cell, leading to increased nitric oxide generation.^42^ Our MR and genetic colocalization analyses support a potential causal role for IGFBP3 on PP in humans, which will require further investigation.

Sclerostin (SOST) was also negatively associated with PP. SOST is a secreted glycoprotein that reduces osteoblastic bone formation by inhibiting canonical Wnt/β-catenin signaling.^43^ Consequently, the increased availability of sclerostin may disrupt the Wnt signaling cascade. Previous studies have shown that Wnt signaling pathways play a role in various processes related to vascular aging.^43^ These pathways have also been linked to arterial stiffness in African ancestry men, a high-risk population for hypertensive disease.^44^ Moreover, observational studies in humans have revealed a correlation between elevated serum SOST levels and carotid intima media thickness, vascular calcification, and arterial stiffness.^45,46^ Genetic studies in humans have demonstrated that variants associated with reduced arterial sclerostin expression were linked to an increased risk of cardiovascular events.^45^ Our findings suggest a causal role for sclerostin on PP regulation, and further studies should be performed to assess whether it represents a suitable therapeutic target to reduce aortic wall calcification and stiffness. Importantly, SOST also regulates bone formation and resorption. An important implication of our findings relates to the current availability of an FDA-approved monoclonal antibody SOST inhibitor for the treatment of osteoporosis (romosozumab), as well as the ongoing development of novel small molecule inhibitors.^47^ Interestingly, romosozumab administration was associated with an increased risk of cardiovascular events (cardiac ischemia, heart failure, cerebrovascular events) in the ARCH trial, in which it was compared with alendronate, although this was not seen in the larger placebo-controlled FRAME trial.^48^ Whether PP increases with SOST inhibition, and whether this is related to an increased cardiovascular risk in this context should be examined in future studies.

Furin is a subtilisin/kex2p-like endoprotease enzyme involved in the processing of several precursor proteins,^49^ including the cleaving of proBNP into NT-proBNP. Furin is involved in lipid metabolism, blood pressure regulation, atherosclerotic plaque formation, ^50^ and has recently been implicated in SARS-CoV-2 transmission.^51^ In individuals with type 2 diabetes, furin was shown to be a better predictor of cardiovascular outcomes than BNP.^52^ Mouse model studies have also shown that furin inhibition decreased vascular remodelling and the progression of atherosclerosis, potentially through the modulation of matrix metallopeptidase-2.^53^ Additionally, Mitchell *et al*. identified the *FURIN* gene locus as a susceptibility locus for arterial stiffness, measured by carotid-femoral pulse wave velocity, in the Framingham study.^54^ Our MR and colocalization analyses highlighted a positive association between genetically predicted furin levels and increased pulse pressure, the mechanism of which remains unknown. Whether furin inhibition leads to a decrease in arterial stiffness and subsequent end-organ damage in humans should be further investigated.

Our analyses highlighted potential putative causal effects of several inflammatory proteins on PP, including TNFSF12, IL-1β, SMOC2 amongst others. Our findings are in line with previous epidemiological and animal studies, that have highlighted roles for IL-1 and TNFα include the regulation of vascular structure and function, potentially through the inhibition of subendothelial release of nitric oxide, and increased endothelin-1 release^55^. Excessive activation of the TNFSF12 pathway, which is upstream of TNFα, has been associated with chronic inflammation, fibrosis and angiogenesis.^56^ IL-1β and TNFα were also shown to be higher in individuals with higher LAS measured by pulse wave velocity.^57^ SPARC-related modular calcium binding (SMOC2) encodes a secreted modular protein that is involved in the regulation of cell-matrix interactions and is implicated in preclinical studies as a contributor to the regulation of fibrotic disorders, potentially through the modulation of inflammatory pathways. It has been suggested that SMOC2 plays a role in tissue remodeling, as it is found to be upregulated in injured aortic vessel wall in animal studies. SMOC2 inhibition was associated with the mitigation of vascular smooth muscle cells proliferation, migration and extracellular matrix degradation.^58^ Our findings suggest that SMOC2 may be a candidate target for aortic tissue remodeling and a therapeutic strategy for LAS and its downstream consequences. Our findings encourage further investigation of inflammatory biomarkers such as TNFSF12 and IL-1β as therapeutic targets for atherosclerosis-independent conditions, including the mitigation of increased PP and LAS risk.

### Strengths and Limitations

Our study has notable strengths. Given the high power and comprehensive proteomics platform used to conduct our analyses, our study provided a unique opportunity to identify and report multiple novel proteins associated with PP. To our knowledge, this is the largest study to comprehensively investigate the associations of 2,923 proteins with PP, and their interactions with sex, in the UK Biobank, as well as their putative causal effect as assessed using an MR framework. The combination of observational analyses and state-of-the-art genetic epidemiology methods increase the robustness of our findings. Nonetheless, our findings should be interpreted in the context of their limitations. Notably, the study was conducted in a predominantly European ancestry population, thereby precluding generalization to other ancestry groups. Considering the reported ethnic disparities in the development of LAS, whereby individuals of African descent are reported to exhibit more premature vascular aging compared to individuals of European descent, ^59,60^ extending proteomic and genomic research to diverse populations is crucial to inform the development of more effective and equitable therapeutic strategies. Furthermore, our observational analyses were limited to participants of the UK Biobank with a mean age of 56-57 years, resulting in a larger proportion of postmenopausal women. Since menopause may increase age-related LAS and PP, this age limitation may have biased association estimates between plasma protein levels and PP. As a result, we may have overlooked relevant proteins that were not considered in MR analyses. Future studies should therefore consider a broader set of proteins in a more age-inclusive population group. Additionally, SV is an important determinant of PP in addition to arterial compliance. The development and general availability of well-validated genetic instruments for carotid-femoral pulse wave velocity (the reference metric of large artery stiffness) should be pursued for this purpose. Whereas we adjusted for SV when appropriate, we note that PP is influenced by the left ventricular ejection pattern, in addition to arterial compliance. Moreover, not all compliance in the arterial system is provided by large arteries, and therefore we cannot exclude some contribution of medium-size (muscular artery) compliance to our findings.

Furthermore, MR analyses rely on several assumptions that impact the reliability of causal inference, which we aimed to overcome through a series of complementary methods. Firstly, we restricted MR analyses to *cis*-acting genetic instruments to limit horizontal pleiotropy. We also implemented colocalization analyses as sensitivity to test whether MR assumptions were violated at each locus. Of note, the number of genetic instruments and instrument strength varied across proteins. Instruments with more variants are favoured in terms of statistical power to detect genetic protein-disease associations, potentially leading to more precise estimates. We still ensured that the genetic instruments employed for the MR analyses were robust and validated our findings using sensitivity analyses. Lastly, MR instruments are used as a proxy of lifelong genetic effects and the MR thereby assumes time-fixed effects, which may differ from pharmacologic interventions that may occur over shorter time periods with different potency. Our analyses assume linearity in the genetic model and linearity in the protein-to-outcome effect. Violations of these assumptions could lead to inefficiencies but are unlikely to generate false-positive results Further investigation, including more specific drug target analyses and validation in model systems is required to establish the causal mechanisms and effects of protein inhibition or stimulation on PP. Prioritized candidate therapeutic targets remain to be evaluated in experimental studies.

## Conclusions

Our study identifies multiple novel proteins with a putative causal effect on PP and unravelled biological sex differences in the manifestation of PP. Notably, our findings identify NPPB with a high level of statistical confidence, which could have important implications given the current availability of FDA-approved medications to boost NPPB effects. Our findings will lead to further investigations regarding the biology and clinical role of the identified candidate therapeutic targets.

## Supplementary Material

Supplementary data

Supplementary tables

## Figures and Tables

**Table 1:** Baseline characteristics of UK Biobank Pharma Proteomics Project (UKB-PPP) included in this study (N=53,016)

	Participants (N=53,016)
**Age at blood draw, y**	56.80 ± 8.21
**Female, N**	28,581 (53.89%)
**Body mass index, kg/m^2^**	27.46 ± 4.78
**Stroke volume, mL**	115.65 ± 37.16
**Blood pressure, mmHg**	
Systolic blood pressure	139.66 ± 19.73
Diastolic blood pressure	82.09 ± 10.73
Mean arterial pressure	105.12 ± 13.08
Pulse pressure	57.57 ± 14.96
**Smoking status, N**	
Never	28,672 (54.06%)
Previous	18,489 (34.87%)
Current	5,600 (10.56%)
**Ethnicity, N**	
Asian	1,134 (2.14%)
Black	704 (1.33%)
White	49,437 (93.22%)
Mixed	349 (0.66%)
Other	539 (1.02%)
**Medication use, N**	
Cholesterol-lowering medication	6,054 (11.41%)
Antihypertensive medication	6,423 (12.11%)
**Blood biochemistry**	
Total cholesterol (mmol/L)	4.52 ± 0.96
LDL cholesterol (mmol/L)	1.69 ± 0.44
HDL cholesterol (mmol/L)	1.29 ± 0.32
Triglycerides (mmol/L)	1.47 (1.04 to 2.14)
Creatinine (umol/L)	0.06 ± 0.01

Continuous variables are summarized as mean ± standard deviation or median (interquartile range). Categorical variables are summarized as N (%).

**Table 2:** List of top 20 proteins associated with pulse pressure with a corrected *P*-value <0.001 in the UK Biobank.

Protein Name	Symbol	Associations with PP	
Model 1 (adjusted for age, sex, BMI, and MAP)		β (mmHg)	Standard Error
N-terminal pro b-type natriuretic peptide	NTPROBNP	0.103	0.003
Natriuretic peptide B	NPPB	0.075	0.003
Angiopoietin 2	ANGPT2	0.068	0.003
Renin	REN	0.063	0.003
Gastric inhibitory peptide	GIP	0.053	0.004
Collagen type IV alpha 1 chain	COL4A1	0.053	0.003
SPARC/Osteonectin, Cwcv And Kazal Like Domains Proteoglycan 1	SPOCK1	-0.044	0.003
Phospholipase A2 group VII	PLA2G7	-0.045	0.003
Tumor necrosis factor superfamily member 10	TNFSF10	-0.045	0.003
Oligodendrocyte myelin glycoprotein	OMG	-0.043	0.003
Collagen triple helix repeat containing 1	CTHRC1	0.053	0.004
Plasminogen activator, tissue type	PLAT	-0.047	0.004
Brevican	BCAN	-0.043	0.003
Cluster of differentiation 93	CD93	0.042	0.003
C-type lectin domain containing 14A	CLEC14A	0.041	0.003
Thrombospondin 2	THBS2	0.04	0.003
Peptide YY	PYY	0.043	0.003
Repulsive Guidance Molecule BMP Co-Receptor A	RGMA	0.039	0.003
C-X-C motif chemokine ligand 10	CXCL10	-0.04	0.003
Low-density lipoprotein receptor	LDLR	-0.037	0.003
Apolipoprotein M	APOM	-0.037	0.003
Cluster of differentiation 276	CD276	0.036	0.003
**Model 2 (adjusted for age, sex, BMI, MAP and SV)**			
N-terminal pro b-type natriuretic peptide	NTPROBNP	0.082	0.010
Brevican	BCAN	-0.059	0.009
C-X-C motif chemokine ligand 10	CXCL10	-0.055	0.009
Myelin oligodendrocyte glycoprotein	MOG	-0.055	0.009
Angiopoietin 2	ANGPT2	0.05	0.009
Oligodendrocyte myelin glycoprotein	OMG	-0.048	0.009
Plasminogen Activator, Tissue Type	PLAT	-0.055	0.010
Natriuretic peptide B	NPPB	0.049	0.009
Leptin	LEP	-0.079	0.015
Thrombospondin-2	THBS2	0.05	0.009
Kallikrein related peptidase	KLK6	-0.046	0.009
Protein tyrosine phosphatase receptor type N2	PTPRN2	-0.047	0.009
Interleukin 1 receptor antagonist	IL1RN	-0.054	0.011
Phospholipase A2 group 10	PLA2G10	0.044	0.009
Retbindin	RTBDN	-0.044	0.009
Histamine N-Methyltransferase	HNMT	-0.046	0.010
Collagen type IV alpha 1 chain	COL4A1	0.043	0.009
Cluster of differentiation 38	CD38	-0.052	0.011
TAFA chemokine like family member 5	TAFA5	-0.045	0.01
Synaptotagmin 1	SYT1	-0.049	0.011
SPARC/Osteonectin, Cwcv And Kazal Like Domains Proteoglycan 1	SPOCK1	-0.039	0.009

The top 20 proteins that were significantly associated with pulse pressure for two linear regression models were selected for this table. Proteins were sorted by *P*-value.

**Table 3:** Protein associations with pulse pressure from Mendelian randomization and linear regression analyses. Proteins that are shown in this table were significantly associated with pulse pressure in Mendelian randomization with an adjusted *P*-value<0.05 after false discovery rate (FDR) correction. Effect estimates from observational analyses are also shown.

*Name*	*Category*	*Associations with pulse pressure from* *linear regression analyses*				*Mendelian randomization analyses*			
		β (mmHg)	SE	*q*	N_SNPs_	β (mmHg)	Lower 95%CI	Upper 95%CI	*q*
***NPPB***	Cardiometabolic	0.075	0.004	<0.001	1	-1.404	-1.850	-0.957	<0.001
***IGFBP3***	Cardiometabolic	-0.037	0.004	<0.001	3	-1.143	-1.573	-0.713	<0.001
***EFEMP1***	Cardiometabolic	0.040	0.004	<0.001	2	1.626	0.744	2.508	0.012
***LTBP2***	Cardiometabolic	0.037	0.004	<0.001	1	-1.634	-2.420	-0.848	0.003
***COMP***	Cardiometabolic	0.026	0.004	<0.001	4	0.755	0.381	1.129	0.005
***KLKB1***	Inflammation	-0.026	0.004	<0.001	3	-0.405	-0.618	-0.191	0.008
***SMOC2***	Inflammation	0.023	0.004	<0.001	6	-0.413	-0.621	-0.206	0.005
***PRSS53***	Neurology	-0.021	0.004	<0.001	9	-0.162	-0.255	-0.069	0.024
***SOST***	Cardiometabolic	-0.021	0.004	<0.001	1	-2.459	-3.947	-0.971	0.0420
***FURIN***	Oncology	-0.020	0.004	<0.001	1	1.309	0.883	1.734	<0.001
***TNFSF12***	Inflammation	-0.016	0.003	<0.001	1	0.453	0.220	0.687	0.007
***F13B***	Inflammation	-0.015	0.004	<0.001	6	-0.408	-0.601	-0.216	0.002
***FGF5***	Inflammation	0.014	0.004	<0.001	8	0.479	0.340	0.618	<0.001
***CD46***	Cardiometabolic	0.013	0.004	<0.001	1	-2.722	-3.742	-1.703	<0.001
***SLC9A3R2***	Oncology	0.014	0.004	0.001	1	1.575	0.788	2.363	0.005
***SMAD5***	Oncology	-0.013	0.004	0.002	1	-3.694	-5.721	-1.667	0.013
***UBE2L6***	Cardiometabolic	-0.013	0.004	0.002	1	-0.669	-1.020	-0.318	0.008
***ABO***	Inflammation	0.012	0.004	0.006	8	0.217	0.145	0.289	<0.001
***ADAMTS8***	Oncology	0.010	0.003	0.010	5	-0.598	-0.908	-0.287	0.008
***ITGAL***	Inflammation	-0.011	0.004	0.020	1	3.742	1.442	6.042	0.045
***IL1B***	Inflammation	-0.010	0.003	0.020	1	3.770	1.472	6.069	0.043
***ADM***	Oncology	0.010	0.004	0.046	1	1.271	0.745	1.798	<0.001

**Abbreviations:**ABO, Alpha 1-3-N-Acetylgalactosaminyltransferase and alpha 1-3-galactosyltransferase**;**ADM, adrenomedullin**;**ADAMTS8, A disintegrin and metalloproteinase with thrombospondin motifs 8; CD46, cluster of differentiation 46; COMP: cartilage oligomeric matrix protein; EFEMP1, EGF-containing fibulin-like extracellular matrix protein 1; IL1B, interleukin 1B; F13B, coagulation factor XIII B subunit; ITGAL, integrin alpha-L; KLKB1, kallikrein B1; LTBP2, Latent Transforming Growth Factor Beta Binding Protein 2; PRSS53, serine protease 53; SLC9A3R2, ; SMAD5, SMAD Family Member 5; SMOC2, SPARC Related Modular Calcium Binding 2; SOST: sclerostin; TNFSF12, TNF superfamily member 12; UBE2L6, ubiquitin conjugating enzyme E2 L6

## Clinical Perspectives

- Our study identified hundreds of novel proteins associated with pulse pressure, that may serve as biomarkers of large artery stiffness (LAS). Our causal inference analyses identified 16 proteins with a putative causal effect on pulse pressure, warranting further investigation for their potential role as therapeutic candidates.
- The identification of novel biomarkers of pulse pressure highlights the importance of conducting proteomic screening and triangulating evidence from observational, genetic and clinical evidence. This approach may guide in identifying novel therapeutic avenues and may facilitate the early diagnosis of large artery stiffness and downstream target organ damage.
- Notably, our study identified natriuretic peptide B (NPPB) to be potentially causally associated with pulse pressure with a high level of statistical confidence. This may have important implications given the current availability of FDA-approved medications to boost NPPB effects.

## References

[1] Kanasi E, Ayilavarapu S, Jones J (2016). The aging population: demographics and the biology of aging. Periodontol 2000.

[2] Tang B, Li Z, Hu S, Xiong J (2022). Economic Implications of Health Care Burden for Elderly Population. Inquiry.

[3] Jones CH, Dolsten M (2024). Healthcare on the brink: navigating the challenges of an aging society in the United States. NPJ Aging.

[4] Kaess BM, Rong J, Larson MG, Hamburg NM, Vita JA, Levy D, Benjamin EJ, Vasan RS, Mitchell GF (2012). Aortic Stiffness, Blood Pressure Progression, and Incident Hypertension. JAMA.

[5] Chirinos JA, Segers P, Hughes T, Townsend R (2019). Large-Artery Stiffness in Health and Disease: JACC State-of-the-Art Review. Journal of the American College of Cardiology.

[6] Chirinos JA, Segers P, Hughes T, Townsend R (2019). Large-Artery Stiffness in Health and Disease: JACC State-of-the-Art Review. J Am Coll Cardiol.

[7] Niiranen TJ, Kalesan B, Mitchell GF, Vasan RS (2019). Relative contributions of pulse pressure and arterial stiffness to cardiovascular disease: The framingham heart study. Hypertension.

[8] Said MA, Eppinga RN, Lipsic E, Verweij N, van der Harst P (2018). Relationship of Arterial Stiffness Index and Pulse Pressure With Cardiovascular Disease and Mortality. Journal of the American Heart Association.

[9] Townsend RR, Wilkinson IB, Schiffrin EL, Avolio AP, Chirinos JA, Cockcroft JR, Heffernan KS, Lakatta EG, McEniery CM, Mitchell GF (2015). Recommendations for Improving and Standardizing Vascular Research on Arterial Stiffness: A Scientific Statement From the American Heart Association. Hypertension.

[10] Yasuno S, Ueshima K, Oba K, Fujimoto A, Hirata M, Ogihara T, Saruta T, Nakao K (2010). Is pulse pressure a predictor of new-onset diabetes in high-risk hypertensive patients?: a subanalysis of the Candesartan Antihypertensive Survival Evaluation in Japan (CASE-J) trial. Diabetes care.

[11] Zheng M, Zhang X, Chen S, Song Y, Zhao Q, Gao X, Wu S (2020). Arterial Stiffness Preceding Diabetes: A Longitudinal Study. Circ Res.

[12] Cohen JB, Mitchell GF, Gill D, Burgess S, Rahman M, Hanff TC, Ramachandran VS, Mutalik KM, Townsend RR, Chirinos JA (2022). Arterial Stiffness and Diabetes Risk in Framingham Heart Study and UK Biobank. Circ Res.

[13] Chirinos JA (2020). Large Artery Stiffness and New-Onset Diabetes. Circ Res.

[14] Domanski M, Norman J, Wolz M, Mitchell G, Pfeffer M (2001). Cardiovascular risk assessment using pulse pressure in the first national health and nutrition examination survey (NHANES I. Hypertension.

[15] Ferkingstad E, Sulem P, Atlason BA, Sveinbjornsson G, Magnusson MI, Styrmisdottir EL, Gunnarsdottir K, Helgason A, Oddsson A, Halldorsson BV (2021). Large-scale integration of the plasma proteome with genetics and disease. Nat Genet.

[16] Sun BB CJ, Traylor M, Benner C, Hsu YH, Richardson TG (2022). Genetic regulation of the human plasma proteome in 54,306 UK Biobank participants. bioRxiv.

[17] Pietzner M, Wheeler E, Carrasco-Zanini J, Cortes A, Koprulu M, Worheide MA, Oerton E, Cook J, Stewart ID, Kerrison ND (2021). Mapping the proteo-genomic convergence of human diseases. Science.

[18] Eldjarn GH, Ferkingstad E, Lund SH, Helgason H, Magnusson OT, Gunnarsdottir K, Olafsdottir TA, Halldorsson BV, Olason PI, Zink F (2023). Large-scale plasma proteomics comparisons through genetics and disease associations. Nature.

[19] Davies NM, Holmes MV, Davey Smith G (2018). Reading Mendelian randomisation studies: a guide, glossary, and checklist for clinicians. BMJ.

[20] Skrivankova VW, Richmond RC, Woolf BAR, Davies NM, Swanson SA, VanderWeele TJ, Timpson NJ, Higgins JPT, Dimou N, Langenberg C (2021). Strengthening the reporting of observational studies in epidemiology using mendelian randomisation (STROBE-MR): explanation and elaboration. BMJ.

[21] Au Yeung SL, Gill D (2023). Standardizing the reporting of Mendelian randomization studies. BMC Med.

[22] Sudlow C, Gallacher J, Allen N, Beral V, Burton P, Danesh J, Downey P, Elliott P, Green J, Landray M (2015). UK biobank: an open access resource for identifying the causes of a wide range of complex diseases of middle and old age. PLoS Med.

[23] Sun BB, Chiou J, Traylor M, Benner C, Hsu YH, Richardson TG, Surendran P, Mahajan A, Robins C, Vasquez-Grinnell SG (2023). Plasma proteomic associations with genetics and health in the UK Biobank. Nature.

[24] Martins D, Tareen N, Pan D, Norris K (2002). The relationship between body mass index and pulse pressure in older adults with isolated systolic hypertension. Am J Hypertens.

[25] Wang YY, Yang S, Chen SW (2023). Relationship between body mass index and pulse pressure in a non-diabetic population: evidence from a multicenter, cross-sectional study. Eur Rev Med Pharmacol Sci.

[26] Benjamini Y, Hochberg Y (1995). Controlling the False Discovery Rate: a Practical and Powerful Approach to Multiple Testing. Journal of the Royal Statistical Society.

[27] Sun BB, Maranville JC, Peters JE, Stacey D, Staley JR, Blackshaw J, Burgess S, Jiang T, Paige E, Surendran P (2018). Genomic atlas of the human plasma proteome. Nature.

[28] Palmer TM, Lawlor DA, Harbord RM, Sheehan NA, Tobias JH, Timpson NJ, Davey Smith G, Sterne JA (2012). Using multiple genetic variants as instrumental variables for modifiable risk factors. Stat Methods Med Res.

[29] Hemani G, Zheng J, Elsworth B, Wade KH, Haberland V, Baird D, Laurin C, Burgess S, Bowden J, Langdon R (2018). The MR-Base platform supports systematic causal inference across the human phenome. Elife.

[30] Burgess S, Butterworth A, Thompson SG (2013). Mendelian randomization analysis with multiple genetic variants using summarized data. Genet Epidemiol.

[31] Harvey A, Montezano AC, Lopes RA, Rios F, Touyz RM (2016). Vascular Fibrosis in Aging and Hypertension: Molecular Mechanisms and Clinical Implications. Can J Cardiol.

[32] Azzo JD, Dib MJ, Zagkos L, Zhao L, Wang Z, Chang CP, Ebert C, Salman O, Gan S, Zamani P (2024). Proteomic Associations of NT-proBNP (N-Terminal Pro-B-Type Natriuretic Peptide) in Heart Failure With Preserved Ejection Fraction. Circ Heart Fail.

[33] Yambe M, Tomiyama H, Koji Y, Motobe K, Shiina K, Gulnisia Z, Yamamoto Y, Yamashina A (2006). B-Type Natriuretic Peptide and Arterial Stiffness in Healthy Japanese Men. American Journal of Hypertension.

[34] Chen YC, Lee MC, Lee CJ, Ho GJ, Yin WY, Chang YJ, Hsu BG (2013). N-terminal pro-B-type natriuretic peptide is associated with arterial stiffness measured using the cardio-ankle vascular index in renal transplant recipients. J Atheroscler Thromb.

[35] Levy D, Hwang S-J, Kayalar A, Benjamin EJ, Vasan RS, Parise H, Larson MG, Wang TJ, Selhub J, Jacques PF (2007). Associations of Plasma Natriuretic Peptide, Adrenomedullin, and Homocysteine Levels With Alterations in Arterial Stiffness. Circulation.

[36] Liu Q, Dang A-m, Chen B-w, Lv N-q, Wang X, Zheng D-y (2015). N-terminal Pro-B-type Natriuretic Peptide is Associated with Arterial Stiffness as Measured According to the Brachial-ankle Pulse Wave Velocity in Patients with Takayasu Arteritis. Journal of Atherosclerosis and Thrombosis.

[37] Mitchell GF, Solomon SD, Shah AM, Claggett BL, Fang JC, Izzo J, Abbas CA, Desai AS, Martinez-Castrillon M, Gables C (2021). Hemodynamic Effects of Sacubitril-Valsartan Versus Enalapril in Patients With Heart Failure in the EVALUATE-HF Study. Circulation: Heart Failure.

[38] Myhre PL, Prescott MF, Murphy SP, Fang JC, Mitchell GF, Ward JH, Claggett B, Desai AS, Solomon SD, Januzzi JL (2022). Early B-Type Natriuretic Peptide Change in HFrEF Patients Treated With Sacubitril/Valsartan: A Pooled Analysis of EVALUATE-HF and PROVE-HF. JACC Heart Fail.

[39] Rutten JHW, Mattace-Raso FUS, Verwoert GC, Lindemans J, Hofman A, Witteman JCM, van den Meiracker AH (2010). Arterial stiffness as determinant of increased amino terminal pro-B-type natriuretic peptide levels in individuals with and without cardiovascular disease – the Rotterdam Study. Journal of Hypertension.

[40] Kim HS (2013). Role of insulin-like growth factor binding protein-3 in glucose and lipid metabolism. Ann Pediatr Endocrinol Metab.

[41] DeStefano AL, Larson MG, Mitchell GF, Benjamin EJ, Vasan RS, Li J, Corey D, Levy D (2004). Genome-Wide Scan for Pulse Pressure in the National Heart, Lung and Blood Institute’s Framingham Heart Study. Hypertension.

[42] Kielczewski JL, Jarajapu YP, McFarland EL, Cai J, Afzal A, Calzi Li, Chang KH, Lydic T, Shaw LC, Busik J (2009). Insulin-like growth factor binding protein-3 mediates vascular repair by enhancing nitric oxide generation. Circ Res.

[43] Catalano A, Bellone F, Morabito N, Corica F (2020). Sclerostin and Vascular Pathophysiology. Int J Mol Sci.

[44] Kuipers AL, Miljkovic I, Barinas-Mitchell E, Nestlerode CS, Cvejkus RK, Wheeler VW, Zhang Y, Zmuda JM (2020). Wnt Pathway Gene Expression Is Associated With Arterial Stiffness. J Am Heart Assoc.

[45] Golledge J, Thanigaimani S (2022). Role of Sclerostin in Cardiovascular Disease. Arteriosclerosis, Thrombosis, and Vascular Biology.

[46] Figurek A, Rroji M, Spasovski G (2020). Sclerostin: a new biomarker of CKD-MBD. Int Urol Nephrol.

[47] Yu S, Li D, Zhang N, Ni S, Sun M, Wang L, Xiao H, Liu D, Liu J, Yu Y (2022). Drug discovery of sclerostin inhibitors. Acta Pharm Sin B.

[48] Reid IR (2022). What is the risk of cardiovascular events in osteoporotic patients treated with romosozumab?. Expert Opin Drug Saf.

[49] Nakayama K (1997). Furin: a mammalian subtilisin/Kex2p-like endoprotease involved in processing of a wide variety of precursor proteins. Biochem J.

[50] Ren K, Jiang T, Zheng XL, Zhao GJ (2017). Proprotein convertase furin/PCSK3 and atherosclerosis: New insights and potential therapeutic targets. Atherosclerosis.

[51] Xiang Q, Wu J, Zhou Y, Li L, Tian M, Li G, Zhang Z, Fu Y (2024). SARS-CoV-2 Membrane protein regulates the function of Spike by inhibiting its plasma membrane localization and enzymatic activity of Furin. Microbiol Res.

[52] Fathy SA, Abdel Hamid FF, Zabut BM, Jamee AF, Ali MA, Abu Mustafa AM (2015). Diagnostic utility of BNP, corin and furin as biomarkers for cardiovascular complications in type 2 diabetes mellitus patients. Biomarkers.

[53] Yakala GK, Cabrera-Fuentes HA, Crespo-Avilan GE, Rattanasopa C, Burlacu A, George BL, Anand K, Mayan DC, Corliano M, Hernandez-Resendiz S (2019). FURIN Inhibition Reduces Vascular Remodeling and Atherosclerotic Lesion Progression in Mice. Arterioscler Thromb Vasc Biol.

[54] Mitchell GF, DeStefano AL, Larson MG, Benjamin EJ, Chen MH, Vasan RS, Vita JA, Levy D (2005). Heritability and a genome-wide linkage scan for arterial stiffness, wave reflection, and mean arterial pressure: the Framingham Heart Study. Circulation.

[55] Ayhan H, Kasapkara HA, Aslan AN, Durmaz T, Keles T, Akcay M, Akar Bayram N, Bastug S, Bilen E, Sari C (2015). Relationship of Neutrophil-to-Lymphocyte Ratio with Aortic Stiffness in Type 1 Diabetes Mellitus. Can J Diabetes.

[56] Burkly LC (2014). TWEAK/Fn14 axis: the current paradigm of tissue injury-inducible function in the midst of complexities. Semin Immunol.

[57] Tuttolomondo A, Pecoraro R, Butta C, Di Raimondo D, Ferrante A, Della Corte V, Ciccia F, Bellia C, Giardina A, Raffa A (2015). Arterial stiffness indexes and serum cytokine levels in seronegative spondyloarthritis: relationships between stiffness markers and metabolic and immunoinflammatory variables. Scand J Rheumatol.

[58] Wang X, Wang M, Zhou Z, Zou X, Song G, Zhang Q, Zhou H (2023). SMOC2 promoted vascular smooth muscle cell proliferation, migration, and extracellular matrix degradation by activating BMP/TGF-beta1 signaling pathway. J Clin Biochem Nutr.

[59] Schutte AE, Kruger R, Gafane-Matemane LF, Breet Y, Strauss-Kruger M, Cruickshank JK (2020). Ethnicity and Arterial Stiffness. Arterioscler Thromb Vasc Biol.

[60] Rogers RG, Onge JM (2005). Race/ethnic and sex differentials in pulse pressure among us adults. Ethn Dis.

