# Supplementary material for: Proteome-wide Mendelian randomization identifies natriuretic peptide-B and novel proteins as potential regulators of pulse pressure in humans
Source: J Am Heart Assoc. Author manuscript; Available in PMC 2025 Sep 2. (PMC7618058; doi:10.1161/JAHA.124.037596)
Supplement: Supplementary data [file EMS208207-supplement-Supplementary_data.docx]

**Proteome-wide Mendelian randomization identifies natriuretic peptide-B and novel proteins as potential regulators of pulse pressure in humans**

Marie-Joe Dib, PhD^1,2^; Devendra Meena, DPhil^3^; James Yarmolinsky, PhD^3^; Joe David Azzo, MD^2^; Oday Salman, MD^2^; Hamed Tavolinejad, MD^1,2^; Sushrima Gan, PhD^4^; Cameron Beeche, BS^1,2^; Bianca Pourmussa, BS^1,2^; Dipender Gill, MD, PhD^3^; Stephen Burgess, PhD^5,6^; Julio A. Chirinos, MD, PhD^1,2^

^1^Division of Cardiovascular Medicine, Hospital of the University of Pennsylvania, Philadelphia PA

^2^ University of Pennsylvania Perelman School of Medicine, Philadelphia, PA

^3^Department of Epidemiology and Biostatistics, School of Public Health, Imperial College London, UK.

^4^Department of Pediatrics (Cardiology) and Cardiovascular Institute, Stanford University

^5^MRC Integrative Epidemiology Unit, University of Bristol, Bristol, UK.

^6^ Department of Public Health and Primary Care, University of Cambridge, Cambridge, UK.

**Supplemental Methods**

***Pathway enrichment analyses***

Associations between proteins and pulse pressure (*P*<0.05) were taken forward to pathway analyses using Ingenuity Pathway Analysis software (Qiagen; Hilden, Germany;www.qiagen.com/ingenuity). The totality of proteins included in the Olink panel was used as the reference set. The analysis calculates a *P*-value (Fisher exact test) quantifying the overlap, and a *z*-score quantifying the likelihood and direction (upregulated or downregulated), between the plasma proteomics pattern and known canonical pathways. Statistical significance was defined as a corrected 2-tailed *P*-value<0.05.

***Genome-wide association study of pulse pressure***

We defined and calculated PP as the difference between systolic and diastolic blood pressure for participants of the UK Biobank. We filtered the population to include individuals who were self-reported British, had no genetic kinship to other participants, had no sex chromosome aneuploidy and poor heterozygosity/missingness rates. We excluded participants with proteomics data from this subset as the aim is to utilize these summary statistics for two-sample MR analyses, avoiding sample overlap between exposure (*i.e.,* protein levels) and outcome (*i.e.,* pulse pressure) datasets. This resulted in the inclusion of 230,422 participants for genetic association analyses. We then conducted a GWAS of PP using PLINK v2.0^18^, adjusting for sex, age, and the first 10 genetic principal components (PCs). Quantitative phenotypes and covariates were standardized. Single nucleotide polymorphisms (SNPs) and individuals with low genotype calls (<98%) were removed. Discrepancies between the recorded sex of the individuals and their sex inferred from X chromosome were checked. SNPs with a low minor allele frequency (MAF) <0.01 and those that deviated from Hardy–Weinberg equilibrium (HWE<1e-10) were excluded.

***Bayesian colocalization analyses***

Upon identifying genetic evidence for proteins with putative causal effects on PP using MR at P_FDR_<0.05, we performed Bayesian colocalization analyses as sensitivity to confirm that each protein and PP shared the same causal variant.^22^ The colocalization method assumes that a maximum of 1 causal variant is present within a given gene locus for a trait of interest, and calculates posterior probabilities for each of the following competing hypotheses: H_0_ (no causal variants), H_1_ (causal variant for trait 1, the protein level in this study), H_2_ (causal variant for trait 2, PP in this study), H_3_ (distinct causal variants for traits 1 and 2), and H_4_ (shared causal variants for traits 1 and 2, supporting a causal association). A high posterior probability for H_4_ suggests colocalization, supporting MR results. In this study, we considered a posterior probability for H_4_ > 0.50 as the threshold for evidence of colocalization. A high posterior probability for H_3_ suggests that the MR findings may be confounded by linkage disequilibrium (LD) between genetic instruments. Priors were calibrated to p_1_ (probability that the variant is associated with protein levels only), and p_2_ (probability that the variant is associated with PP only) at 10^-4^, and p_12_ (probability that an arbitrary variant is associated with the trait for both protein levels and pulse pressure) at 10^−5^. We also performed sensitivity analyses by varying the value of p*_12_* from 10⁻⁴ to 10⁻⁸ while keeping p*_1_* and p*_2_* constant at 10⁻⁴.

***Software***

Analyses were performed using R version 4.0.3. Regression models for proteome-wide observational analyses were implemented using the **lm()** function. We used the **MendelianRandomization** (version 0.6.0) and **TwoSampleMR** (version 0.5.6) packages in R to perform all MR analyses. Colocalization was performed using **Coloc** (version 3.2-1).

**Supplemental Figures**

**Figure S1. Canonical pathway analyses of proteins associated with pulse pressure in (A) Model 1, adjusted for age, sex, body mass index and mean arterial pressure (B) Model 2, adjusted for age, sex, body mass index, mean arterial pressure and stroke volume.** FDR corrected *P* values of 0.05 was used to determine significance. Numbers indicate the Z-score corresponding to the direction and strength of each association.

**(A)**

**
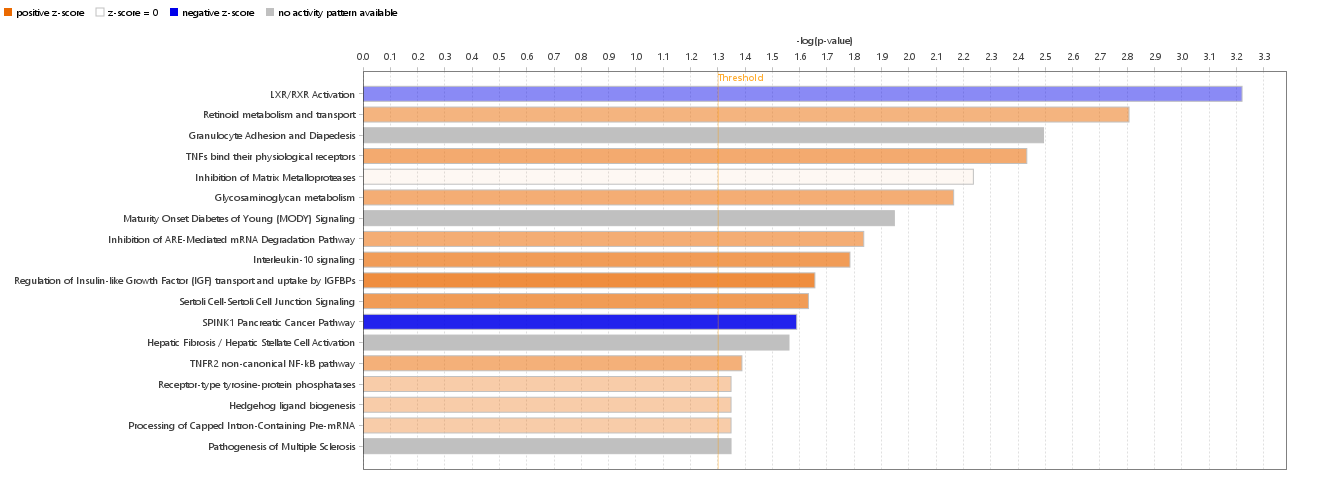
**

**(B)
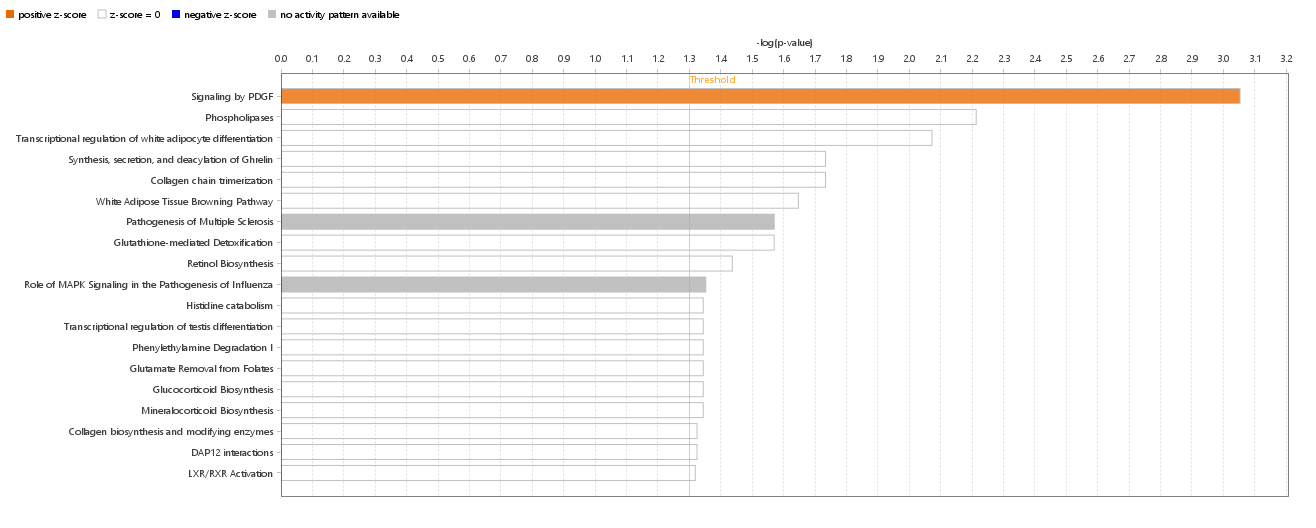
**

**Figure S2. Volcano plots representing sex-stratified proteins associations with pulse pressure for females (A) and males (B). Linear regression model was adjusted for age, body mass index (BMI), and mean arterial pressure (MAP).** The plot shows beta estimates against the false discovery rate (FDR) corrected log-10 *p* value, to better visualize the importance of each biomarker in order of significance. The dashed line represents the 5% FDR alpha threshold.

**(A)**

**
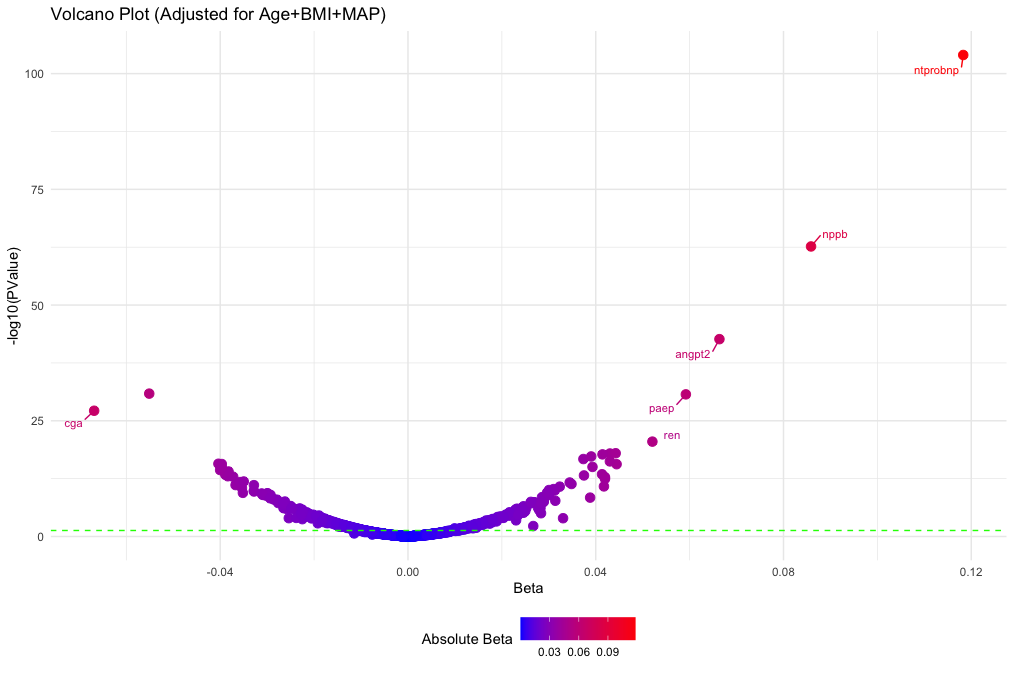
**

**(B)**

**
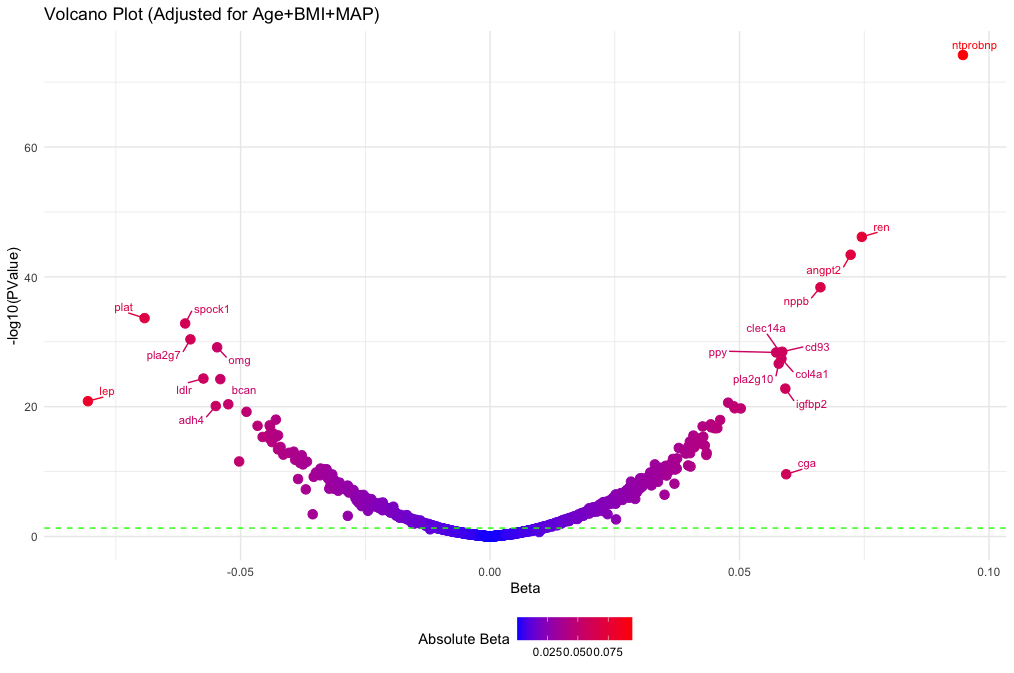
**

**Figure S3. Canonical pathway analyses of proteins with differential associations between males and females for pulse pressure.** The regression model was adjusted for age, sex, body mass index and mean arterial pressure. FDR corrected *P* values of 0.05 was used to determine significance. Numbers indicate the Z-score corresponding to the direction and strength of each association.

**
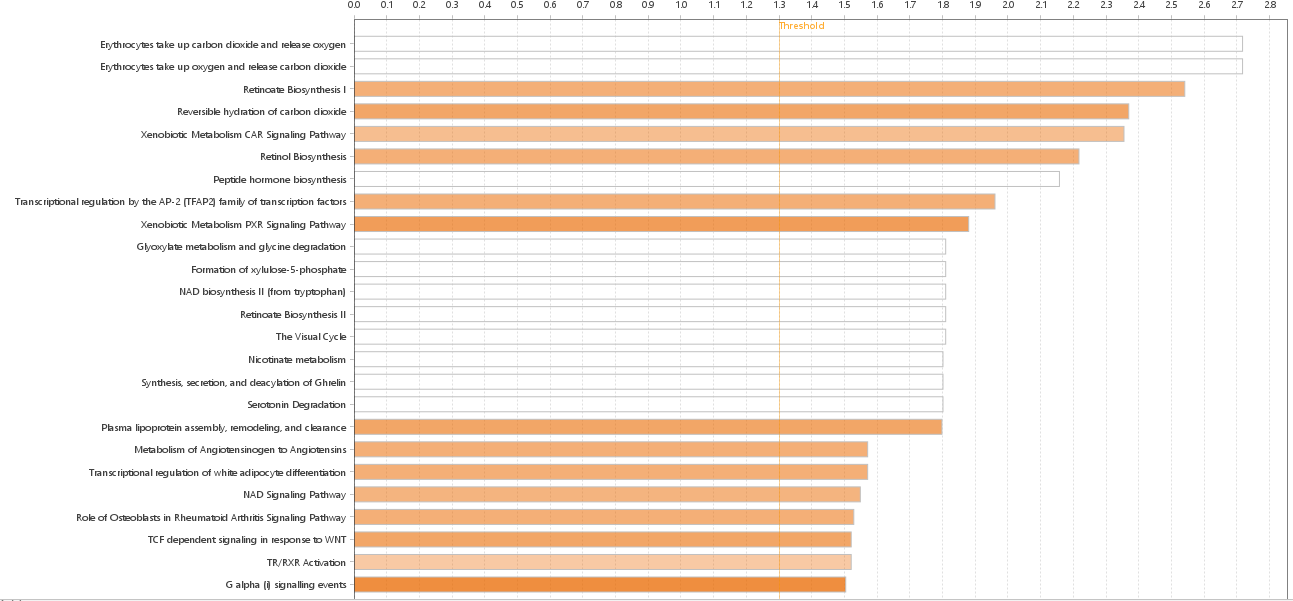
**

**Figure S4. Volcano plot representing proteins significantly associated with pulse pressure adjusted for antihypertensive medication use in Mendelian randomization analyses.** The plot shows β estimates against the false discovery rate (FDR) corrected log-10 *p* value, to better visualize the importance of each biomarker in order of significance. The dashed line represents the 5% FDR alpha threshold.

**
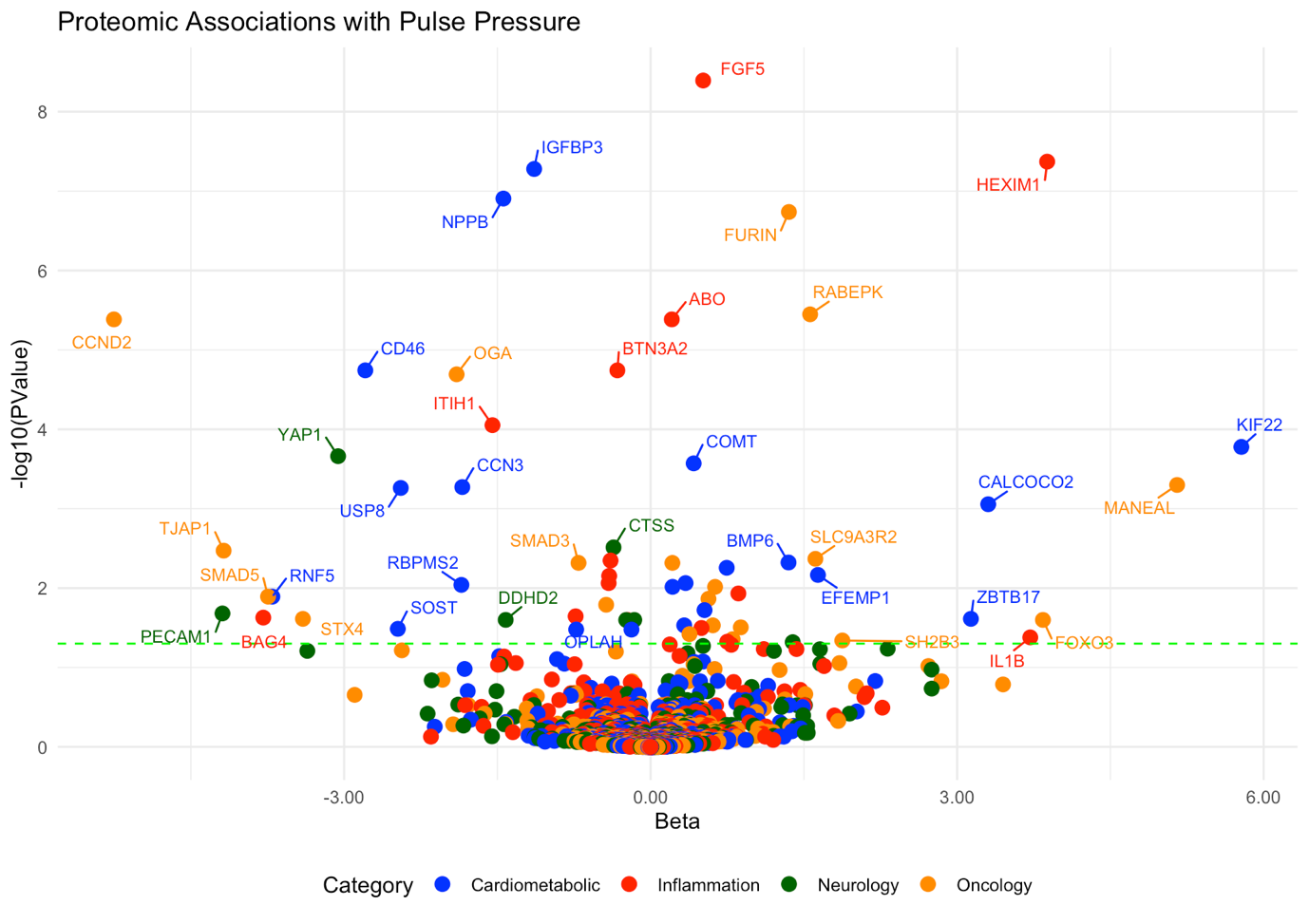
**

**Figure S5. Sensitivity of coloc results to the choice of prior *p_12_* on the shared causal variant hypothesis for all proteins identified as associated with pulse pressure in Mendelian randomization analyses.**

**ABO ADAMTS8**

**
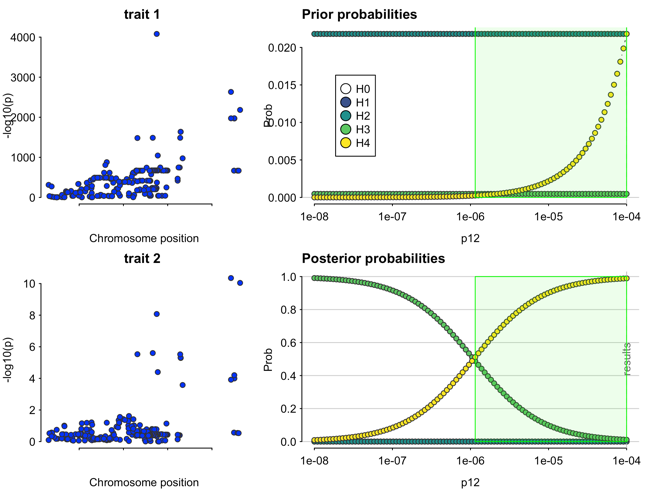

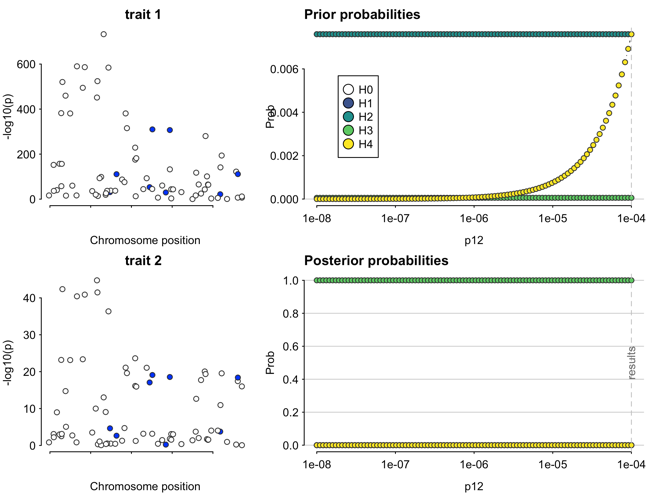
**

**ADM CD46**

**
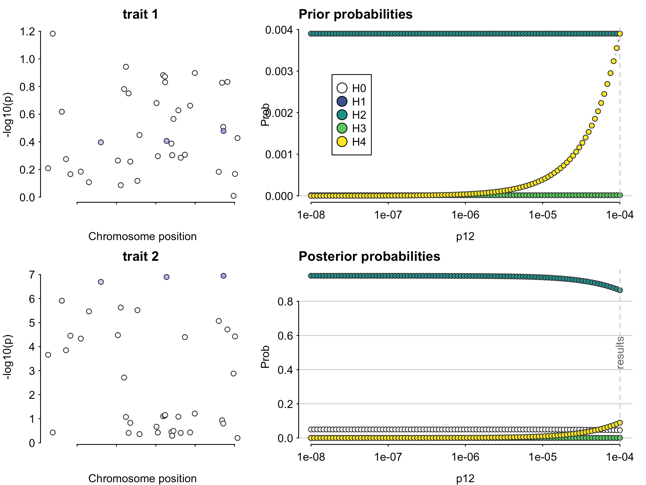

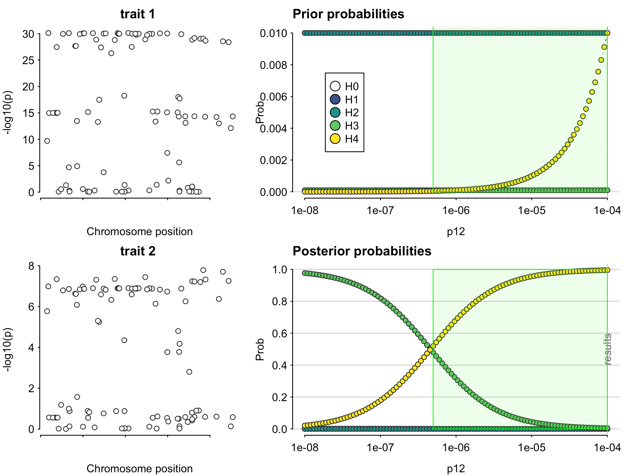
**

**COMP EFEMP1**

**
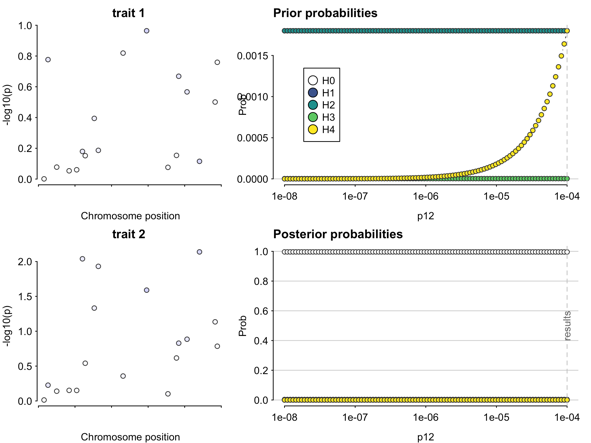

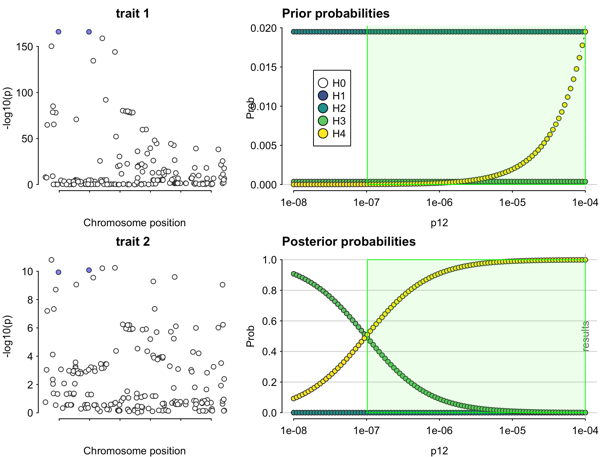
**

**F13B FGF5**

**
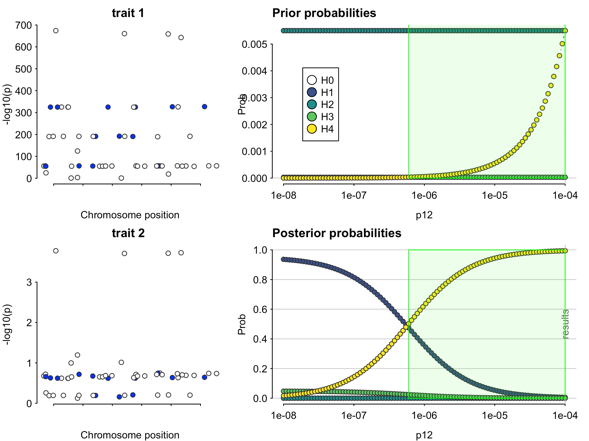

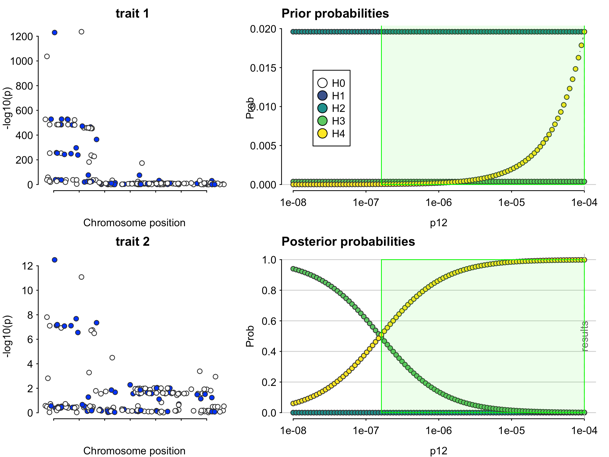
**

**FURIN IGFBP3**

**
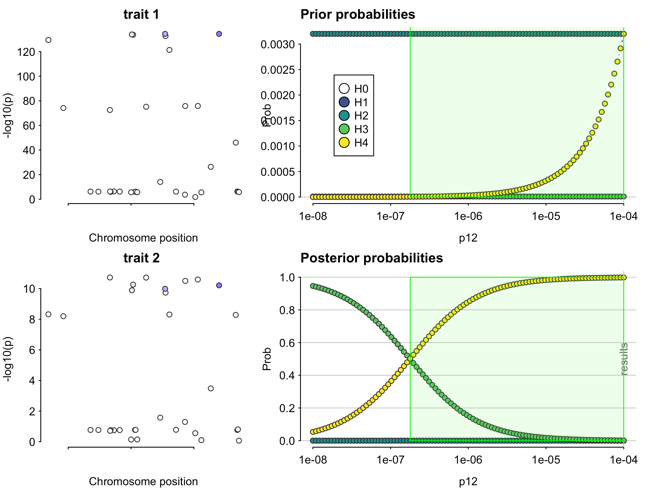

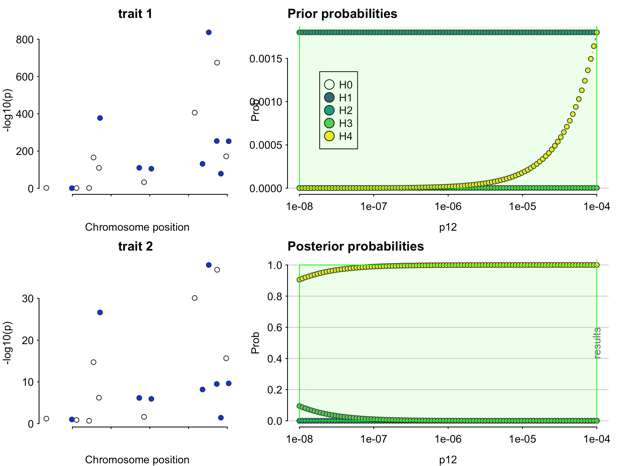
**

**IL1B ITGAL**

**
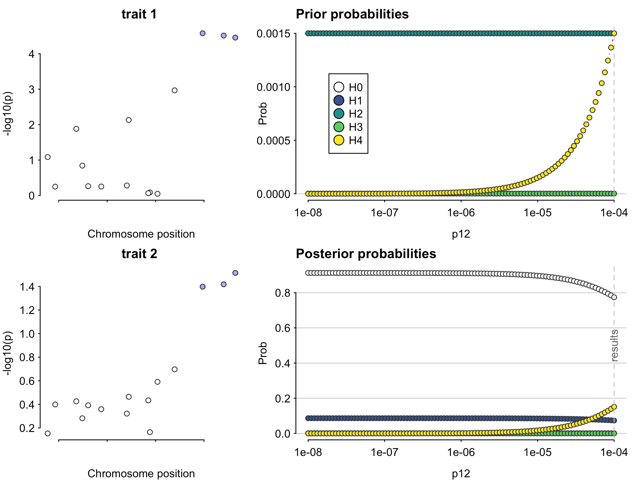

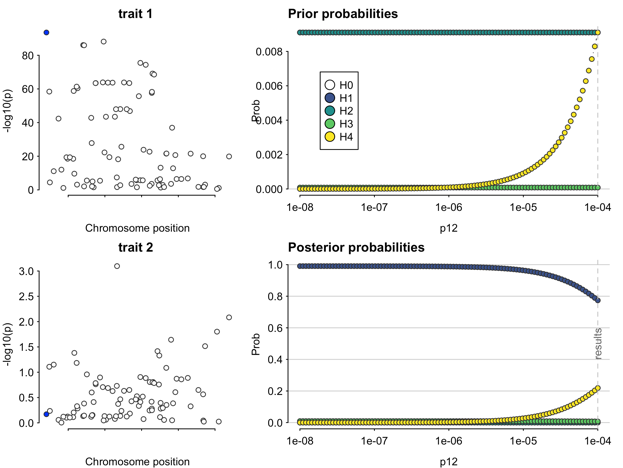
**

**KLKB1 LTBP2**

**
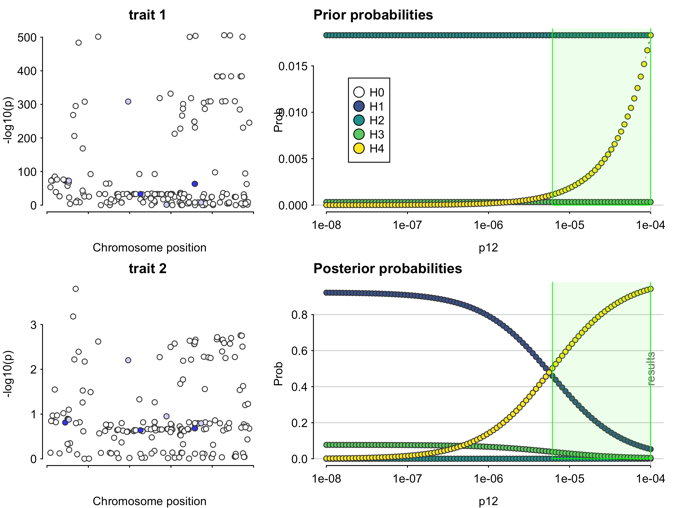

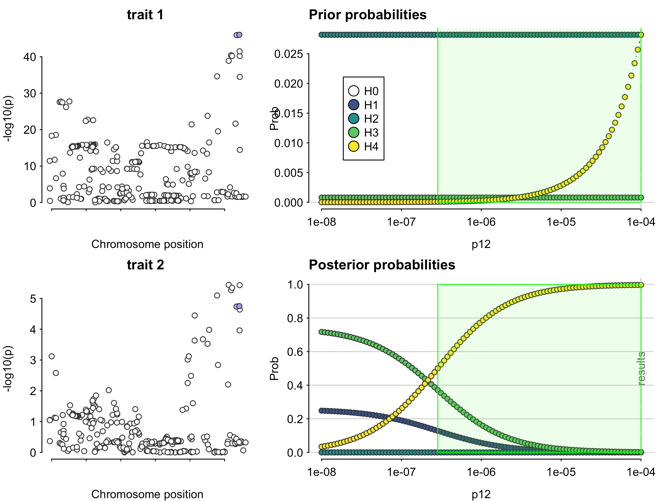
**

**PRSS53 SLC9A3R2**

**
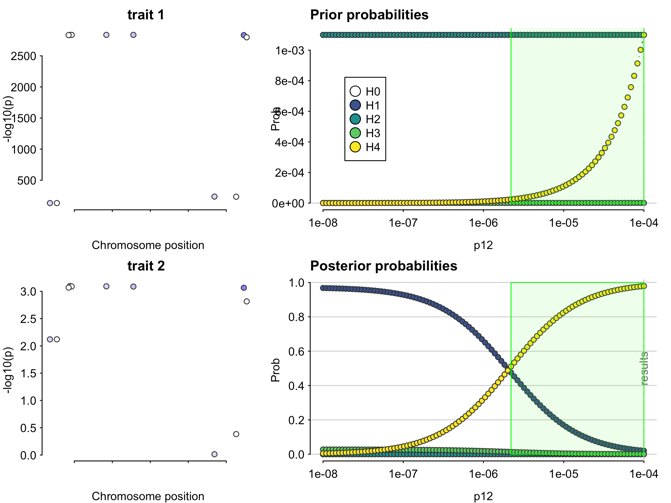

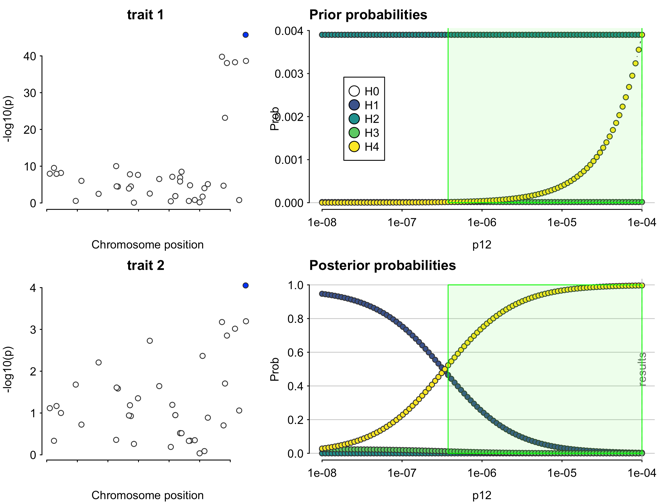
**

**SMAD5 SMOC2**

**
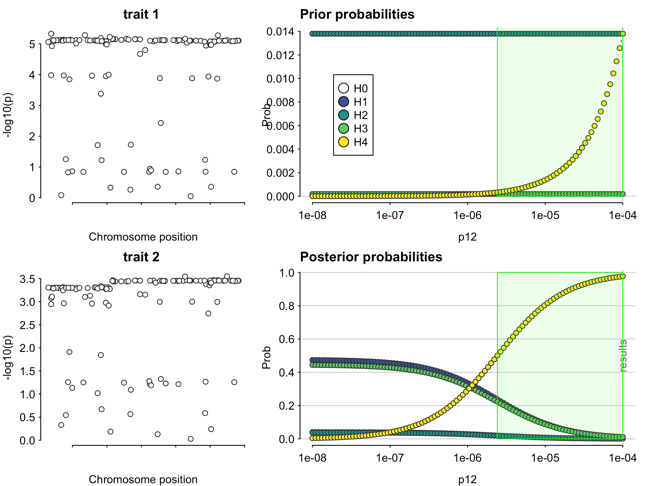

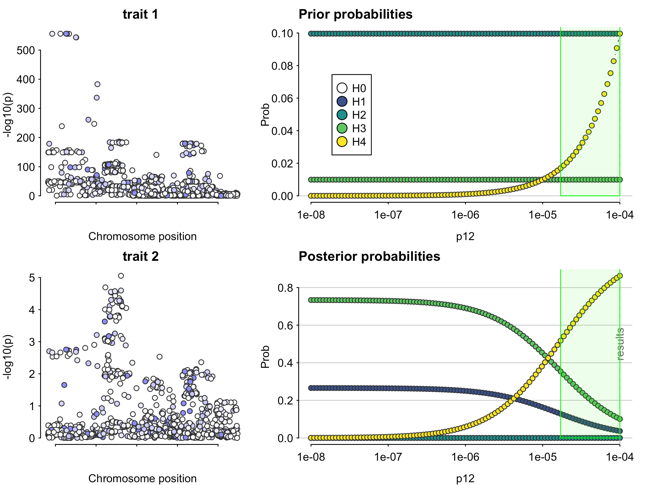
**

**SOST TNFSF12**

**
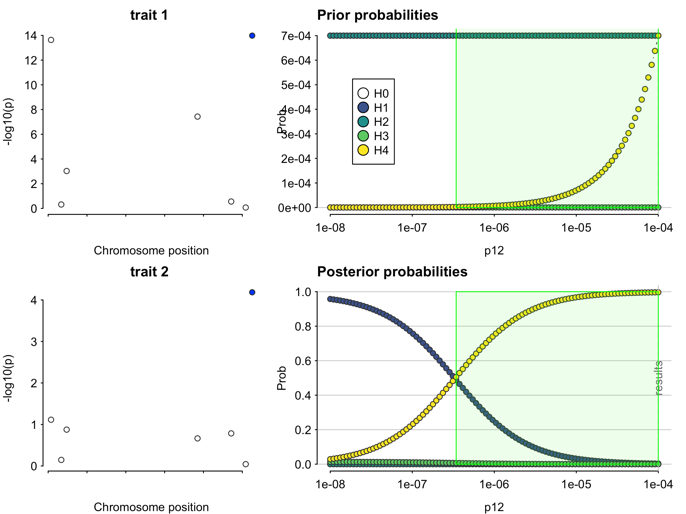

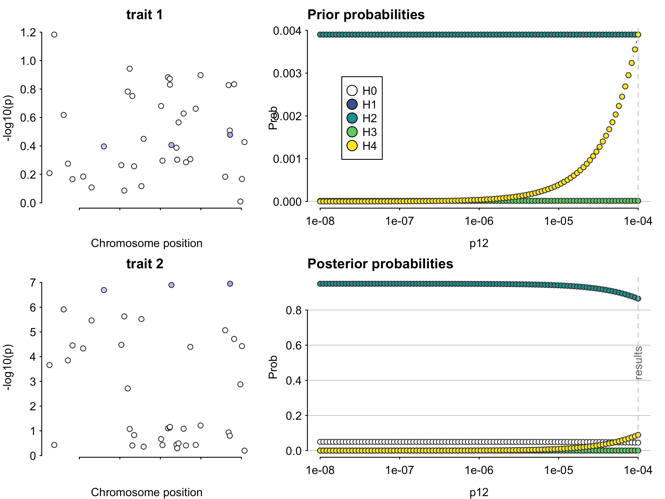
**

**UBE2L6**

**
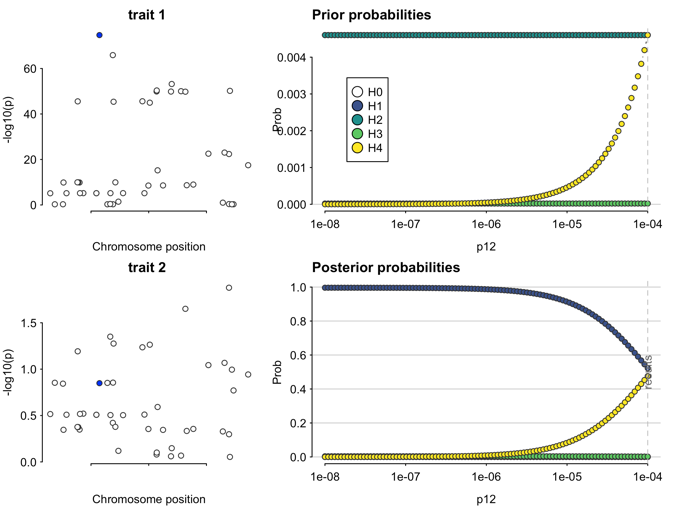
**
